# Identification of tumor‐associated macrophage subsets that are associated with breast cancer prognosis

**DOI:** 10.1002/ctm2.239

**Published:** 2020-12-06

**Authors:** Elisabeth Strack, P. Alexander Rolfe, Annika F. Fink, Katrin Bankov, Tobias Schmid, Christine Solbach, Rajkumar Savai, Weixiao Sha, Leon Pradel, Sylvia Hartmann, Bernhard Brüne, Andreas Weigert

**Affiliations:** ^1^ Faculty of Medicine Institute of Biochemistry I Goethe‐University Frankfurt Frankfurt Germany; ^2^ EMD Serono Research and Development Institute Billerica Massachusetts; ^3^ Dr. Senckenberg Institute of Pathology University Hospital Frankfurt Frankfurt Germany; ^4^ Department of Gynecology University Hospital Frankfurt Frankfurt Germany; ^5^ Max Planck Institute for Heart and Lung Research Bad Nauheim Germany; ^6^ Merck Healthcare KGaA Darmstadt Germany; ^7^ Frankfurt Cancer Institute (FCI) Goethe University Frankfurt Frankfurt Germany; ^8^ Institute of Lung Health (ILH) Justus Liebig University Giessen Germany; ^9^ German Cancer Consortium (DKTK) Partner Site Frankfurt Frankfurt Germany

**Keywords:** breast cancer, flow cytometry, macrophage, transcriptome, tumor microenvironment

## Abstract

**Background:**

Breast cancer is the leading cause of cancer‐related deaths in women, demanding new treatment options. With the advent of immune checkpoint blockade, immunotherapy emerged as a treatment option. In addition to lymphocytes, tumor‐associated macrophages exert a significant, albeit controversial, impact on tumor development. Pro‐inflammatory macrophages are thought to hinder, whereas anti‐inflammatory macrophages promote tumor growth. However, molecular markers to identify prognostic macrophage populations remain elusive.

**Methods:**

We isolated two macrophage subsets, from 48 primary human breast tumors, distinguished by the expression of CD206. Their transcriptomes were analyzed via RNA‐Seq, and potential prognostic macrophage markers were validated by PhenOptics in tissue microarrays of patients with invasive breast cancer.

**Results:**

Normal human breast tissue contained mainly CD206^+^ macrophages, while increased relative amounts of CD206^−^ macrophages were observed in tumors. The presence of CD206^+^ macrophages correlated with a pronounced lymphocyte infiltrate and subsets of CD206^+^ macrophages, expressing SERPINH1 and collagen 1, or MORC4, were unexpectedly associated with improved survival of breast cancer patients. In contrast, MHCII^hi^ CD206^−^ macrophages were linked with a poor survival prognosis.

**Conclusion:**

Our data highlight the heterogeneity of tumor‐infiltrating macrophages and suggest the use of multiple phenotypic markers to predict the impact of macrophage subpopulations on cancer prognosis. We identified novel macrophage markers that correlate with the survival of patients with invasive mammary carcinoma.

## Graphical abstract

- Transcriptome analysis of CD206+ and CD206‐ macrophages reveals differential phenotype.
- CD06+ macrophages coexpressing either MORC4 or SERPINH1 show a positive correlation with patient survival.
- CD206‐ macrophages expressing MHCII are correlated with negative patient survival.

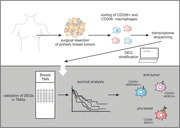

## 1 BACKGROUND

According to the World Health Organization, breast cancer is the leading cause of cancer‐related deaths among women. Current treatment options include surgery, antagonizing estrogen receptor (ER), progesterone receptor (PR), or human epidermal growth receptor 2 (HER2) signaling, as well as radio‐ and chemotherapy. Although overall response rates to breast cancer therapy are encouraging, there are still certain breast cancer subtypes, such as triple‐negative breast cancer (TNBC), that require new therapy options.^1^

The tumor microenvironment primarily consists of a large extent of stromal cells, including immune cells, which are now recognized as critical modulators of tumor development. According to the theory of cancer immunoediting, immune cells are capable of killing tumor cells, particularly in the early stages of tumor development, thereby reducing tumor incidence. In this process immunomodulatory factors such as interleukin (IL)‐10, transforming growth factor β, and immune checkpoint molecules are induced. These factors reprogram recruited immune cells to a tumor‐supportive phenotype.^2^, ^3^ Novel antibody therapies targeting immune checkpoints, such as activation of programmed cell death protein (PD‐1) on lymphoid cells, have shown remarkable results in reactivating antitumor immunity. Unfortunately, only a minority of patients are currently benefiting from this approach.^4^ In addition to lymphocytes, macrophages have also been highlighted as possible targets in tumor immunotherapy since multiple studies have revealed their negative correlation with patient survival.^5^, ^6^, ^7^

Macrophages are highly heterogeneous cells and can be divided into subpopulations based on their functional phenotype.^8^ Pro‐inflammatory macrophages mediate host defense against microbes and are believed to confer antitumor immunity via release of cytokines, such as IL‐12 or tumor necrosis factor α (TNFα). Anti‐inflammatory macrophages are considered tumor‐promoting cells that help to suppress and regulate type 1 inflammatory responses and promote wound healing.^9^, ^10^, ^11^ These subtypes can be distinguished in cancer tissues according to their expression of different markers, including the mannose receptor c‐type 1 (CD206) and the scavenger receptor CD163 for anti‐inflammatory macrophages,^12^ and nitric oxide synthase 2 for inflammatory macrophages.^13^ Current approaches to target tumor‐associated macrophages are centered around macrophage depletion or blocking monocyte recruitment into tumors.^14^ For instance, targeting colony‐stimulating factor 1 (CSF1) receptor with monoclonal antibodies,^15^ or targeting its receptor CSF1R with the FDA‐approved tyrosine kinase inhibitor pexidartinib, which also targets proto‐oncogene receptor tyrosine kinase (KIT) and FMS‐like tyrosine kinase 3 (FTL3),^16^ is utilized to deplete tumor‐associated macrophages in clinical trials. However, specifically targeting tumor‐promoting macrophages, while preserving tumor‐restraining subsets may be a more desirable strategy.

Reliable druggable targets to individually exploit the pro‐ and antitumoral properties of macrophages are still largely elusive. This study aimed to identify novel prognostic macrophage markers in mammary cancer by analyzing the transcriptome of CD206^+^ versus CD206^−^ macrophages, based on the hypothesis that specific subsets within these two macrophage populations are associated with clinical parameters in breast cancer. Potential new subset markers were then validated by multiplex quantitative immunofluorescence, using breast cancer tissue microarrays (TMAs).

## 2 MATERIALS AND METHODS

### 2.1 Reagents

Lipopolysaccharide (LPS) and 3‐[(3‐Cholamidopropyl)dimethylammonio]‐1‐propanesulfonate hydrate (CHAPS) was purchased from Merck (Darmstadt), IL‐4, TNFα, and interferon γ (IFNγ) were from Peprotech (Hamburg), and cyclohexamide (CHX) was obtained from Roth (Karlsruhe).

### 2.2 Sample collection and study approval

The University Cancer Center (UCT) Frankfurt provided the human breast cancer samples and normal tissue samples used in this study (cohort overview in Table S1). Patients with breast cancer were included in the study (ICD10‐Code: C50.9). No exclusion criteria were applied. Sample collection started in January 2017 and ended in March 2019. Normal breast tissue was taken from tissue in proximity to the primary cancer site and was visually checked for absence of tumor cells by a pathologist. Both fresh and formalin fixed paraffin embedded (FFPE) samples were obtained from the same patient simultaneously. To ensure epitope stability for fluorescence‐activated cell sorting (FACS) analysis, the fresh tissue samples were stored at 4°C in RPMI 1640 medium (Thermo Fisher Scientific, Waltham, MA) containing 10 μg/mL CHX for a maximum of 2 hours prior to analysis (Figure S1). Written informed consent was obtained from all patients, and the study was approved by the Institutional Review Boards of the UCT Frankfurt and the Ethics Committee at University Hospital Frankfurt (approval numbers: SGO‐01‐2014 and SGO‐01‐2016). All investigations were performed according to the guidelines of the World Medical Association's Declaration of Helsinki.

### 2.3 FACS analysis and sorting of macrophages from human breast cancer samples

Tumors were dissociated using human Tumor Dissociation Kit and the gentleMACS Dissociator (both Miltenyi Biotec, Bergisch Gladbach) following a standard protocol with slight modifications. Briefly, tissue was cut into 2‐4 mm pieces, and processed with the gentleMACS Dissociator using program h_tumor_01. Samples were then incubated for 15 minutes with enzyme mix provided in the human Tumor Dissociation Kit at 37°C, followed by another application of the h_tumor_01 program. Reduced digestion times compared to the standard protocol were required to maintain cell surface epitope integrity (Figure S1). Cell suspensions were then filtered through 70 μm cell strainers to remove aggregates. For FACS sorting of primary macrophages, single cell suspensions were stained with a FACS panel to distinguish myeloid cell subsets (antibodies listed in Table S2, gating strategy shown in Figure S2). If sample size exceeded 0.5 g, one‐third of the single cell suspension was stained with a FACS panel with lymphoid markers (antibodies listed in Table S2, gating strategy shown in Figure S3). Cell suspensions were filtered through 30 μm cell strainers and diluted with PBS before cell sorting or analysis, respectively. Up to 1000 cells were sorted into PBS containing 3 mM EDTA, 25 mM HEPES, and 2% w/v FCS. Cell sorting and analysis was performed on a BD FACS Aria III Sorter. Samples were analyzed with FlowJo software (Tree Star, Ashland, Wilmington, DE, RRID:SCR_008520). All antibodies and secondary reagents were titrated to determine optimal concentrations. Comp‐Beads (BD Biosciences, Heidelberg) were used for single‐color compensation to create multicolor compensation matrices. Fluorescence minus one controls were used for gating. The instrument calibration was controlled daily using Cytometer Setup and Tracking beads (BD Biosciences, Heidelberg).

### 2.4 RNA Sequencing (RNA‐Seq) of primary human macrophages

mRNA‐isolation and cDNA transcription were performed immediately after sorting using SMART‐Seq v4 Ultra Low Input RNA Kit for Sequencing (Takara, Saint‐Germain‐en‐Laye, France) according to the manufacturer's instructions. cDNA was amplified via 16 PCR cycles, and samples were purified after cDNA and library synthesis with AMPure XP beads for PCR Purification (Beckman Coulter, Brea, CA). For the determination of cDNA content, a Qubit 3.0 Fluorometer in combination with Qubit 1x dsDNA HS Assay Kit (both Thermo Fisher Scientific, Waltham, MA) was used. Fragment sizes were determined using High Sensitivity DNA Kit with 2100 Bioanalyzer (both Agilent, Santa Clara, CA). The Covaris M220 system was used for controlled cDNA shearing (2 minutes at 20°C, peak power 75 W, 20% duty factor, burst cycle set at 200). Library preparation was performed with SMARTer ThruPLEX DNA‐Seq Kit and DNA HT Dual Index Kit – 96N Set A (both Takara, Saint‐Germain‐en‐Laye, France). Four samples at a time were subjected to an indexed single‐read sequencing run with 1 × 75 cycles on an Illumina NextSeq 500 system.

### 2.5 RNA‐Seq quantification, quality control, and differential expression

Transcript‐level expression was quantified using Kallisto software version 0.43.1 or 0.46.1 (RRID:SCR_016582)^17^ against the Ensembl (RRID:SCR_002344) human transcript sequences from release 91. Transcript expression was summed to produce gene‐level expression using custom Python code (RRID:SCR_001658) using the gene symbols included in the Ensembl transcripts FASTA file. Based on prior experience with low input samples from small numbers of cells, sequencing output that captured only a few highly expressed genes was excluded; such samples were typically characterized, then compared to successful samples by high counts at a small number of genes and very low counts at a larger number of genes. Thus, we implemented quality control checking using R software version 3.4.1 or higher that looked at the distribution of read counts across samples. More specifically, we plotted the 90th percentile, 80th percentile, 70th percentile, and 60th percentile read count and then looked for samples, which had a very high 90th percentile count but low counts at the others. Again, based on prior experience, we removed samples which appeared to inadequately cover genes at the 80th percentile or below compared to the remainder of the dataset. Differential expression testing was performed using DESeq2 version 1.22 or higher^18^ using a full project dataset (the two macrophage populations plus additional populations not presented here) to estimate dispersions and then comparing the two macrophage populations. The RNA‐Seq data set has been submitted to the NCBI Sequence Read Archive (PRJNA668813).

### 2.6 Heatmap generation

MORPHEUS matrix visualization and analysis software (https://software.broadinstitute.org/morpheus/) was used for heatmap generation. One minus Pearson's correlation was used for hierarchical clustering of rows.

### 2.7 Gene set enrichment analysis

Gene set enrichment analysis (GSEA) (RRID:SCR_003199) was performed with all genes expressed by both macrophage subsets (10.076) after setting a threshold to remove T and NK cell‐related genes using GSEA software version 4.0.0.

### 2.8 Multiplex immunohistochemistry and immunofluorescence analysis

FFPE tissue sections were provided by the University Cancer Center Frankfurt. Cancer Diagnosis Program (CDP) Breast Cancer Progression Tissue Microarrays were obtained from the Cooperative Human Tissue Network and the Cancer Diagnosis Program, which are funded by the National Cancer Institute (USA). All data concerning the CDP TMA can be found at https://chtn.sites.virginia.edu/cdp-progression-info. Other TMAs were obtained from Tristar (TriStar Technology Group LLC, Washington, D.C.). Specifically, a Triple Negative Breast Cancer Tissue Microarray (catalog number: 69572273), a TMA of tumors from patients with Herceptin Eligible Breast Cancer (catalog number: 69571139), and a TMA of tumors from patients with Relapsed ER+ Breast Cancers (catalog number: 69572075‐1621). All tumor sections were stained with Opal 7‐Color Automation IHC Kits (Akoya Biosciences, Menlo Park, CA) in the BOND‐RX Multiplex IHC Stainer (Leica, Wetzlar). The following primary antibodies were used: anti‐CD163 (abcam, Cambridge, UK, ab182422, RRID:AB_2753196), anti‐CD68 (Agilent, Santa Clara, CA, M0876, RRID:AB_2074844), anti‐CD206 (Cell Signaling, Danvers, MA, 91992S, RRID:AB_2800175), anti‐PLAC8 (Atlas Antibodies, Bromma, Sweden, HPA040465, RRID:AB_10794875), anti‐SERPINH1 (Novus Biologicals, Centennial, CO, NBP1‐97491, RRID:AB_11189509), anti‐MORC4 (Atlas Antibodies, Bromma, Sweden, HPA000395, RRID:AB_1079405), anti‐MHCII (Cell Signaling, Danvers, MA, 68258S), anti‐pan‐cytokeratin (abcam, Cambridge, UK, ab7753, RRID:AB_306047), anti‐collagen I (abcam, Cambridge, UK, ab6308, RRID:AB_305411), anti‐αSMA (Sigma, Darmstadt, F3777, RRID:AB_476977), and anti‐Ki67 (abcam, Cambridge, UK, ab16667, RRID:AB_302459). Nuclei were counterstained with 4′,6‐diamidino‐2‐phenylindole (DAPI) and mounted with Fluoromount‐G (SouthernBiotech, Birmingham, AL). To test epitope stability of all targets, tissue sections were repeatedly subjected to heat‐induced antigen retrieval, and changes in antigen‐dependent signal after staining were monitored.^19^ The order of antibody staining was selected based on this validation process (Figure S1). A Vectra3 imaging system was used to image the slides at 4x and 20x magnification, and inForm 2.4.9 software (both Akoya Biosciences, Menlo Park, CA) was used for subsequent analysis. Briefly, after cell segmentation, individual markers were scored based on thresholds defined by median expression or by using the phenotyping algorithm provided in the inForm software. Mean values were used whenever samples were present in duplicates.

### 2.9 Cell culture

Human macrophages were cultured in RPMI 1640 medium containing 5% AB‐positive human serum (DRK‐Blutspendedienst Baden‐Württemberg‐Hessen, Frankfurt, Germany), 100 U/mL penicillin, and 100 μg/mL streptomycin. MCF‐7 (RRID:CVCL_0031) and T47D (RRID:CVCL_0553) cells were cultured in 175 cm^2^ culture flasks in RPMI 1640 with stable L‐glutamine medium, 100 U/mL penicillin, 100 μg/mL streptomycin, 10% FCS, 1% sodium pyruvate solution, and 1% non‐essential amino acids. MDA‐MB‐231 (RRID:CVCL_0062) and EVSA‐T (RRID:CVCL_1207) cells were cultured in 175 cm^2^ culture flasks in DMEM, 100 U/mL penicillin, 100 μg/mL streptomycin, 10% FCS, and 2 mM L‐glutamine. EFM‐192A cells (RRID:CVCL_1812) were cultured in 175 cm^2^ culture flasks in RPMI 1640 with stable L‐glutamine medium, 100 U/mL penicillin, 100 μg/mL streptomycin, and 20% FCS (all supplements and media by Thermo Fisher Scientific, Waltham, MA). Cells were maintained in a humidified atmosphere of 5% CO_2_/95% air at 37°C.

### 2.10 Generation of human macrophages from Buffy Coats

Human monocytes were differentiated from peripheral blood mononuclear cells (PBMCs) isolated from Buffy Coats provided by DRK‐Blutspendedienst using Ficoll density gradient centrifugation. PBMCs were cultured for 1 hour in RPMI 1640 medium containing 100 U/mL penicillin and 100 μg/mL streptomycin. Afterwards, non‐attached cells were washed off, and the attached monocytes were cultured in RPMI 1640 medium with 5% human serum for 7 days. Fully differentiated macrophages were stimulated with IL‐4 (5 ng/mL) for 48 hours, IFNγ (100 pg/mL) for 24 hours, or LPS (10 ng/mL) for 24 hours to induce macrophage polarization.

### 2.11 RNA isolation and quantitative reverse transcription‐polymerase chain reaction (RT‐PCR)

RNA from macrophages was isolated using the PeqGold protocol (Peqlab Biotechnologie). RNA was transcribed into cDNA using the Maxima First Strand cDNA Synthesis Fermentas Kit (Thermo Fisher Scientific, Waltham, MA). Quantitative RT‐PCR was performed using PowerUp SYBR Green Master Mix on a QuantStudio 5 Real‐Time‐PCR‐System (Thermo Fisher Scientific, Waltham, MA). All primers were commercial QuantiTect primer assays from Qiagen (Hilden).

### 2.12 Gene silencing in primary human macrophages

Fully differentiated macrophages were transfected with either ON‐TARGETplus Human MORC4 siRNA or siGENOME Non‐Targeting siRNA Control Pools (both Horizon) using HiPerFect Transfection Reagent (Qiagen, Hilden) according to the manufacturer's instructions. Twenty‐four hours after transfection macrophages were used for downstream assays.

### 2.13 In‐vitro co‐culture assay

For a co‐culture of macrophages with various breast cancer cell lines, tumor cells were harvested with trypsin‐EDTA, washed with phosphate‐buffered saline (PBS), and suspended in macrophage media. Tumor cells and macrophages were cultured in a 1:1 ratio for up to 96 hours, in which the majority of tumor cells were killed and phagocytosed.^20^ Afterwards, remaining tumor cells were removed by trypsinization, and macrophage gene expression was analyzed by quantitative RT‐PCR.

### 2.14 Caspase 3 activity assay

Apoptosis in primary human macrophages was induced by the addition of CHX (10 μg/mL) and TNFα (10 ng/mL) for 6 hours at 37°C. Cells were washed once with PBS, harvested by scraping, and pelleted via centrifugation (5 minutes, 1000 g, 4°C). Supernatant was removed, and cells were suspended in 200 μl caspase buffer (100 mM HEPES, 10% sucrose, 0.1% CHAPS, 1 mM EDTA, 10 mM DTT). Samples were sonicated for 10 seconds on ice and afterwards centrifuged for 10 minutes at 13 000 g, 4°C. Protein concentration of supernatant was determined using DC protein assay (BioRad, CA). Fifty μg proteins were loaded in duplicates onto a 96‐well plate, followed by the addition of 10 μl CHAPS (10 mM). Ninety‐six‐well‐plate was measured every 5 minutes for 1 hour using Tecan Infinite 200 PRO plate reader with extinction set to 360 nm and emission at 460 nm at 31°C.

### 2.15 Statistical analysis

GraphPad Prism software version 8.4.0 (Graphpad Software Inc, San Diego, CA, RRID:SCR_002798) was used for data analysis. The *P*‐values were calculated using RM one‐way ANOVA with Geisser‐Greenhouse correction and Tukey's multiple comparisons test or Dunnetts's multiple comparisons test as indicated in the figure legends. Patient survival curves were compared using Mantel‐Cox test and Gehan‐Breslow‐Wilcoxon test. Correlation matrix was computed using Pearson correlation. Tests to pre‐determine sample size were not used. Analysis of TMAs was done in a blinded manner, while all other analyses in an unblinded manner.

## 3 RESULTS

### 3.1 CD206^+^ and CD206^−^ macrophages in mammary tumors show distinct functional phenotypes

**FIGURE 1:**
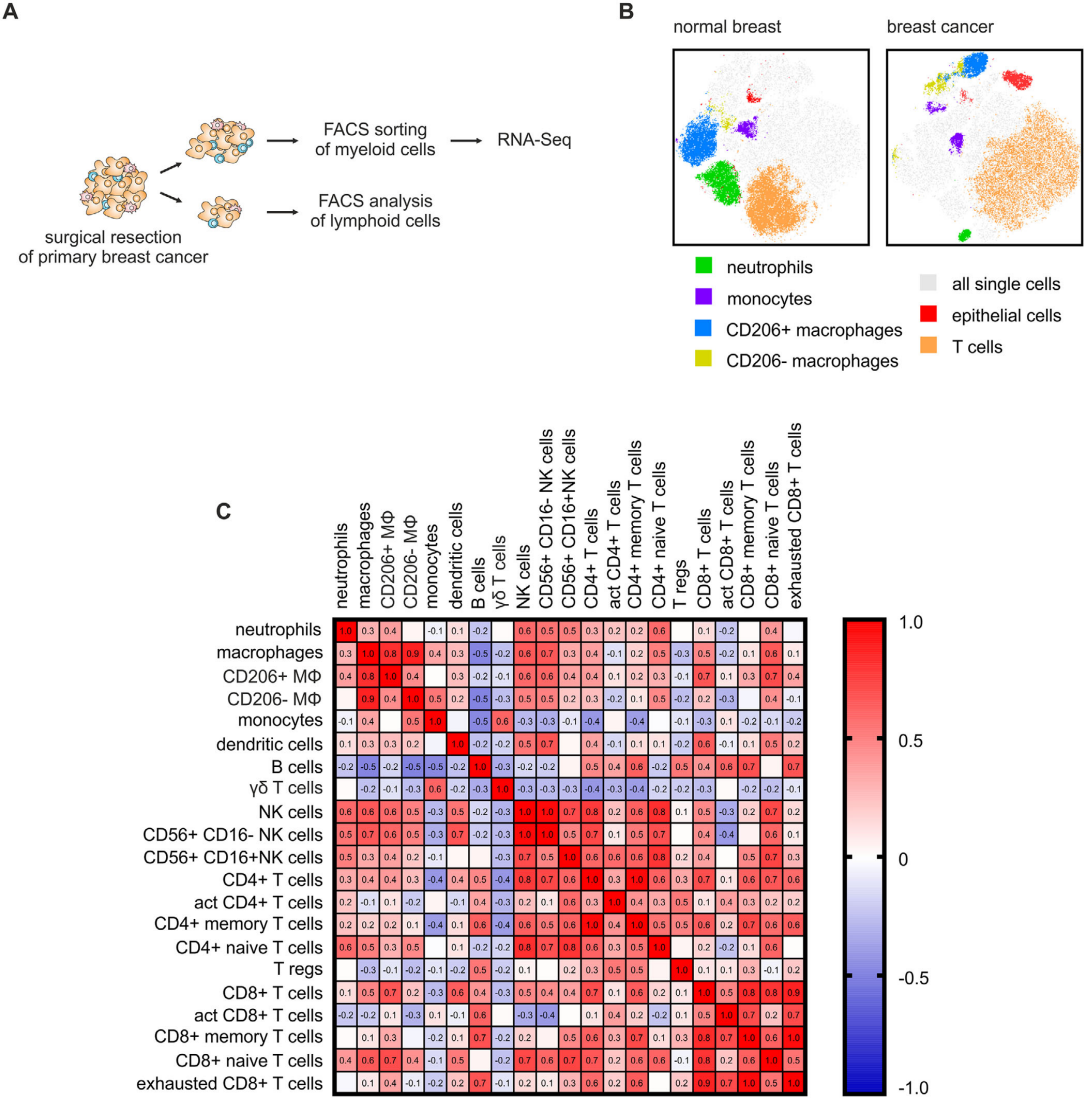
Different macrophage subpopulations in mammary cancer tissue. A, Workflow of mammary tumor tissue analysis. Forty‐eight obtained patient samples were used for FACS sorting and FACS analysis of macrophages and other myeloid cells, while only samples of sufficient size (n = 14) were also used for analyzing lymphoid cell composition. B, tSNE plots of cumulative FACS data from three normal mammary tissues and corresponding breast cancer tissues depicting major cell subsets within all single cells (grey), neutrophils (green), monocytes (violet), CD206+ MΦ (macrophages, blue), CD206− MΦ (yellow), epithelial cells (red), and T cells (orange). C, Correlation matrix of FACS‐analyzed myeloid cells and lymphoid cells. Positive correlation is indicated in red and negative correlation in blue. Numbers indicate Pearson r‐value

We and others previously observed two distinct macrophage populations in primary murine breast tumors, which can be distinguished based on the expression of CD11b, CD11c, and CD206.^21^, ^22^, ^23^, ^24^ CD206^+^ macrophages resembled the resident macrophage population in murine breast tissue, whereas CD206^−^ macrophages increased in abundance during tumor growth in the polyoma middle T oncogene (PyMT) mammary carcinoma model, with both tumor‐infiltrating macrophage populations showing distinct functional properties.^22^, ^23^ Furthermore, in humans we mainly observed CD206^+^ macrophages in untransformed mammary tissue by flow cytometry, while CD206^−^ cells relatively increased in mammary tumors (Figure 1B). Based on these data, we wondered what the phenotypes of these two populations and their impact on mammary tumor prognosis might be. Therefore, we isolated CD206^+^ and CD206^−^ macrophages from 48 patients with mammary carcinoma and explored their phenotype by whole transcriptome RNA‐Seq. First, we used FACS to analyze the myeloid and lymphoid cell composition in these tumors (Figure S4; Figures S2 and S3 for gating strategy). Molecular profiling was done by a trained pathologist. However, we also stained analyzed tumors for common tumor markers (Figure S5). Most samples were from patients diagnosed with Luminal B breast cancer, which is characterized by a poor prognosis, low sensitivity to endocrine therapy and is mostly chemoresistant. Luminal A breast cancer cells have a lower proliferation potential and patients with Luminal A breast cancer generally have a better survival prognosis.^25^ A limited number of samples (n = 6) were obtained from patients with TNBC, whose tumors lack expression of ER, PR, and HER2. When comparing myeloid and lymphoid cell composition with molecular subtypes, we did not observe major alterations in the immune cell composition among molecular breast cancer subtypes in our patient cohort (Figure S4). For the evaluation of macrophage subpopulation‐specific relationships to other immune cells, a FACS data‐based correlation matrix was composed. To generate the matrix, we combined the data from Figure S4. Due to limited sample material only 12 samples had matched myeloid and lymphoid datasets, in the remaining 18 samples only myeloid cells were analyzed. The presence of CD206^+^ macrophages correlated with a pronounced lymphocyte infiltrate, particularly with a high amount of CD8^+^ T cells, which was markedly weaker for CD206^−^ macrophages (Figure 1C). Apparently, the presence of CD206^+^ macrophages correlated with that of potentially tumoricidal lymphocytes.

**FIGURE 2:**
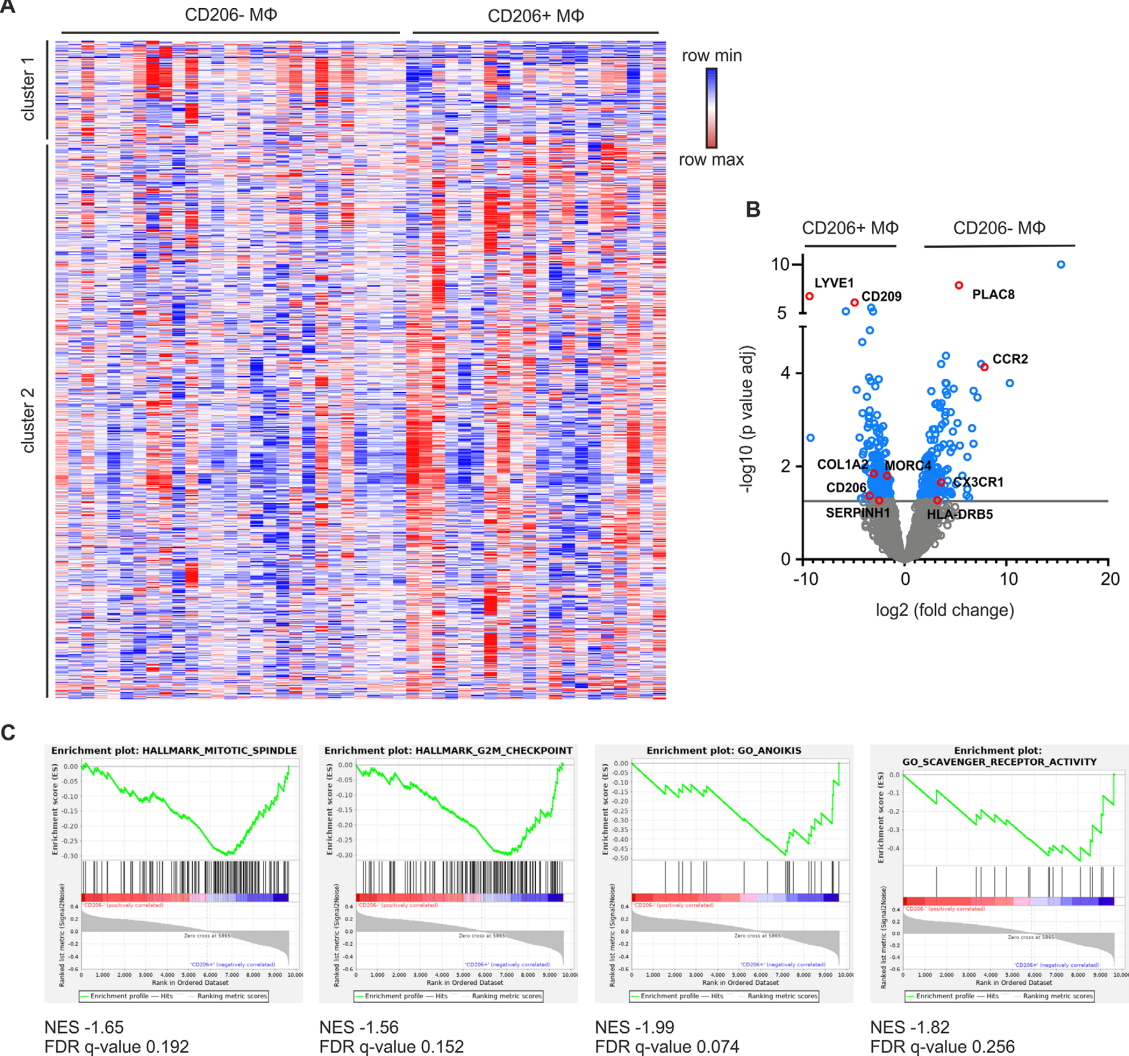
Transcriptome analysis of CD206^+^ and CD206^−^ macrophages. A, Heat‐map representation of all genes after setting a threshold to eliminate lymphocyte‐related genes (10 076 remaining genes) in CD206^−^ MΦ (n = 27) and CD206^+^ MΦ (n = 19). Clustering of rows was performed using one minus Pearson correlation. B, Volcano plot of the relative difference in expression level of all genes (n  =  10,076). DEGs (453) between CD206^−^ and CD206^+^ MΦ are marked in blue. Genes of interest for defining macrophage subpopulations are highlighted in red. C, Representative GSEA plots of enriched pathways in CD206^+^ MΦ when compared to CD206^−^ MΦ. The normalized enrichment score (NES) and false discovery rate (FDR) q‐value are indicated

Next, transcriptomes of the two macrophage subtypes were analyzed by RNA‐Seq using the SMART‐Seq v4 method.^26^ Of the 48 analyzed patient samples, 27 CD206^+^ and 33 CD206^−^ macrophage samples were successfully FACS‐sorted and sequenced, of which 20 CD206^+^ and 27 CD206^−^ macrophage samples passed quality criteria after sequencing (Figure S6 for detailed sample collection). Fifteen of those samples (46.9%) were matched samples from the same patient. Despite an expected high heterogeneity of all samples (Figure S7), DESeq2 analysis resulted in the identification of 453 differentially expressed genes (DEGs) between CD206^+^ and CD206^−^ subpopulations (Figure 2A,B). The cell surface proteins lymphatic endothelium hyaluronan receptor 1 (LYVE1) and CD209 were highly significantly upregulated in CD206^+^ macrophages. In contrast, the expression of major histocompatibility complex (MHC) class II encoding gene HLA‐DRB5, chemokine receptor 2 (CCR2), and C‐X3‐C motif chemokine receptor 1 (CX3CR1) were induced in CD206^−^ macrophages. These markers correspond to two functionally distinct macrophage subsets recently identified in a number of tissues,^27^ further indicating the validity of our approach.

To identify distinct genetic programs defining the two macrophage subsets, we performed GSEA of all DEGs between CD206^+^ and CD206^−^ macrophages (Table S3 and S4). Due to the high sample heterogeneity, there were no significantly (false‐discovery rate <.25) enriched pathways found in CD206^−^ macrophages. However, CD206^+^ macrophages showed enriched genes involved in mitotic spindle formation, G2/M checkpoint control, programmed cell death (anoikis), and scavenger receptor activity (Figure 2C), the former three of those indicating a different turn‐over of CD206^+^ versus CD206^−^ macrophages in human mammary cancer tissue. These findings reveal that CD206^+^ and CD206^−^ macrophages have two distinct phenotypes that correlate with the presence of prognostically relevant lymphocytes, CD8+ T cells, in breast cancer.

### 3.2 Macrophage markers MORC4, SERPINH1, and MHCII correlate with survival in breast cancer

**FIGURE 3:**
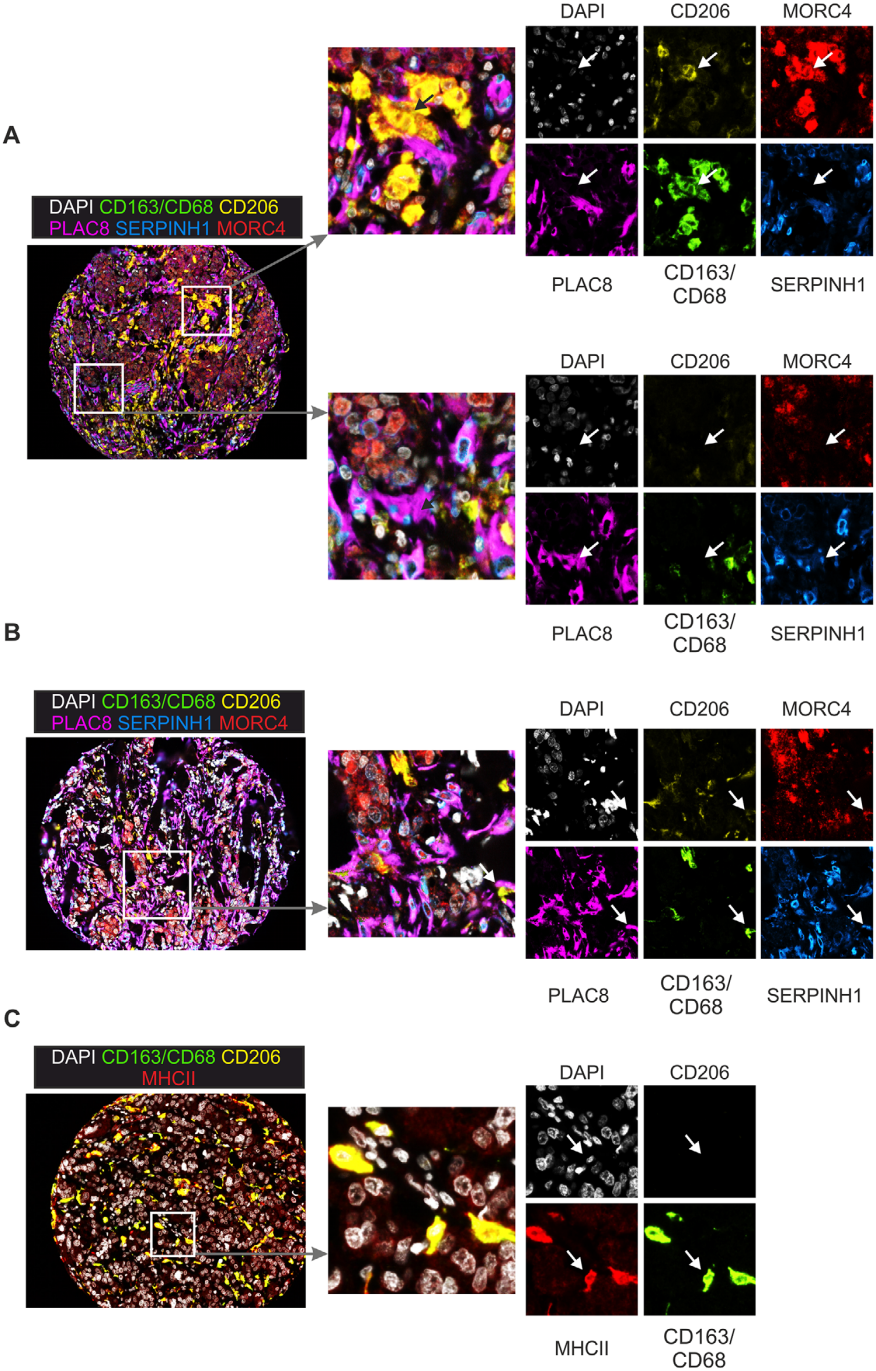
Validation of prognostic macrophage markers. A‐C, Representative immunofluorescence staining of breast cancer TMAs with MΦ markers CD163/CD68 (green) and CD206 (yellow), together with PLAC8 (magenta), SERPINH1 (blue), MORC4 (red) (A, B), or MHCII (red) (C). Nuclei were counterstained with DAPI (white). Magnifications serve to illustrate the presence of CD206^+^ MORC4^+^ (A, upper panels), CD206^+^ SERPINH1^+^ (B), and CD206^−^ MHCII^+^ macrophages (C). Furthermore, SERPINH1^+^ PLAC8^+^ fibroblasts are displayed (A, lower panels)

**FIGURE 4:**
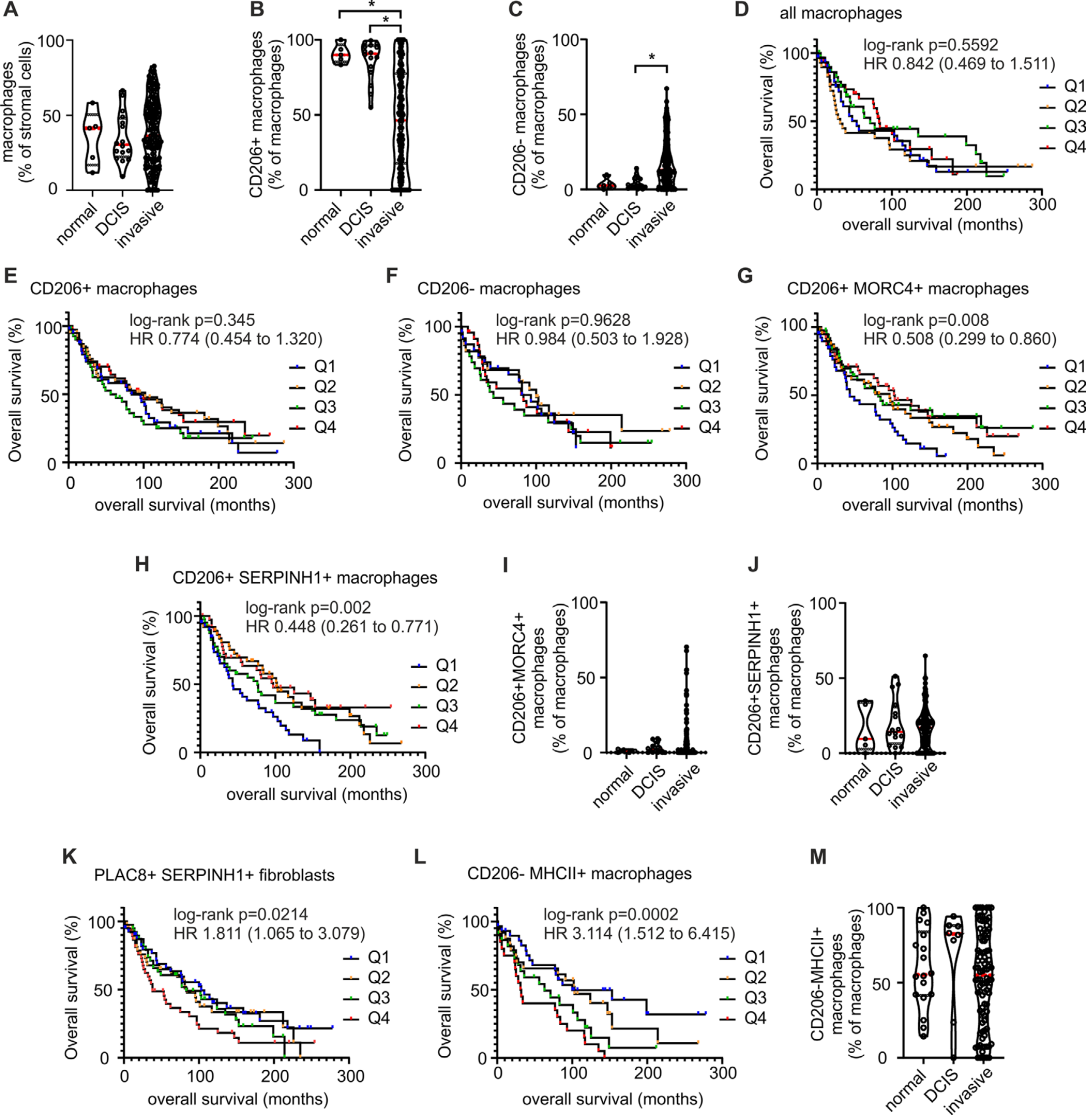
Quantitative analysis of immunofluorescence staining. A‐C, I, J and M, Analysis of TMA staining for the abundance of all macrophages (A) and the CD206^+^ (B) and CD206^−^ (C) subsets in normal tissue (n  =  5), DCIS (n  =  16) and invasive breast cancer (n  =  154). Abundance of CD206+ MORC4+ (I), CD206+ SERPINH1+ (J), and CD206‐ MHCII+ (M) macrophages was also analyzed in the same tissues. *P*‐values were calculated using one‐way ANOVA with Dunn's multiple comparison test. *P < .05. D‐H, K, and L, Kaplan‐Meier estimates analyze the association of indicated cell populations with patient survival. Q1 marks the lowest quartile of indicated cell subtype abundance in tissues and Q4 the highest quartile (n =  38 in each quartile for D‐G, H, and K; n  =  24 for L). Hazard ratios were calculated between Q1 and Q4

Next, we asked if prognostic macrophage markers are contained within our RNA‐Seq dataset. To narrow down the DEGs identified by RNA‐Seq to potentially prognostic markers in CD206^+^ and CD206^−^ macrophages, a filtering strategy using public databases was employed (Figure S6B). We assumed that targets with a robust expression in macrophages (using our RNA‐Seq data), stromal localization (histology data in the Human Protein Atlas^28^), expression in macrophages coexpressing CD206 or not (*BioGPS* ^29^), and predicted clinical significance (*PRECOG database* ^30^) independently of macrophages, would be of particular promise. Since we aimed at validating relevant markers at protein level by multiplex histology, the availability of high‐quality antibodies served as another criterion. After several rounds of testing, the markers MORC4 (MORC family CW‐type zinc finger 4), SERPINH1 (heat shock protein 47) (upregulated in CD206^+^ macrophages), as well as PLAC8 (placenta associated 8), and MHCII (upregulated in CD206^−^ macrophages) followed our selection criteria. Since tumor derived macrophages were not available due to limited numbers of macrophages (typically <1000 cells), the expression of these markers in macrophages was validated by immunofluorescence staining of human monocyte‐derived macrophages (Figure S8). To link patient survival with the presence or absence of these selected markers, TMAs of patients with invasive breast cancer (∼ one‐third of those with distant metastasis) provided by the Cooperative Human Tissue Network and the Cancer Diagnosis Program were analyzed by PhenOptics multiplex histology. Two staining protocols were established. Both included a combination of CD163 and CD68 (labeled with the same fluorochrome) for an unbiased identification of all macrophages (Figure S9), and CD206 to differentiate CD206^+^ from CD206^−^ subpopulations. MORC4, SERPINH1, and PLAC8 were included in the first protocol, while MHCII was included in the second (Figure 3A‐C). Of the total 340 tissue cores contained in the TMA, 154 tissue cores passed the quality criteria for analysis (tissue integrity after staining process, staining quality, autofluorescence) in the first panel, and 94 cores passed in the second panel. Besides invasive mammary carcinoma, the TMAs included tissue samples from normal breast and ductal carcinoma in situ (DCIS), which is a benign form of the mammary carcinoma with intact basement membrane. First, we observed that the number of total macrophages did not dramatically differ between those tissues (Figure 4A), while there was an enrichment of CD206^−^ macrophages in invasive carcinoma compared with DCIS or normal breast tissue (Figure 4B,C), again suggesting CD206^+^ macrophages as the resident mammary macrophage subset. The mere presence of macrophages was not linked to increased survival of the patients in our dataset (Figure 4D), which is in contrast to previous studies.^5^, ^31^, ^32^, ^33^ Also the presence of either CD206^+^ or CD206^−^ macrophages per se did not indicate an association with patient survival (Figure 4E,F). However, macrophages expressing either MORC4 or SERPINH1 together with CD206 were positively correlated with patient survival (Figure 4G,H), although their numbers did not significantly change in invasive carcinoma compared with DCIS or normal breast tissue (Figure 4I,J). In contrast, MORC4 and SERPINH1 expression in CD206^−^ macrophages were not correlated with patient survival (Figures S9A and B). Interestingly, expression of SERPINH1 in fibroblasts, which were spatially related to SERPINH1‐expressing macrophages, and which also expressed PLAC8 (Figure 3A), correlated negatively with patient survival (Figure 4K). PLAC8, which was upregulated in CD206^−^ macrophages on RNA level, showed no correlation with patient survival (data not shown). However, CD206^−^ macrophages expressing MHCII were significantly associated with poor patient survival (Figure 4L), while their abundance did not differ between invasive carcinoma, DCIS, or normal breast tissue (Figure 4M).

To validate our findings, we used other commercially available TMAs of patients with relapsed ER+ breast cancer (80 patients, cores of 54 patients passed quality criteria), HER2+ breast cancer (40 patients, cores of 38 patients passed quality criteria), and TNBC (50 patients, cores of 26 patients passed quality criteria) (Figure S11; Table S5), and performed PhenOptics multiplex histology with our selected markers. In all three TMAs, neither the presence of macrophages per se nor the expression of CD206 was correlated with survival. However, macrophages expressing MORC4 or SERPINH1 together with CD206 were again positively correlated with patient survival, while MHCII expression correlated with poor survival (Figure S11). This correlation appeared to be most pronounced for TNBC (Figure S11C), but the limited sample size precludes premature conclusions of a prominent role of these macrophage subsets in TNBC. Taken together, our data indicate that expression of MORC4, SERPINH1, and MHCII at protein level in combination with CD206 may serve as macrophage markers for survival in mammary carcinoma.

### 3.3 SERPINH1 expression in CD206^+^ macrophages marks collagen‐expressing macrophages

**FIGURE 5:**
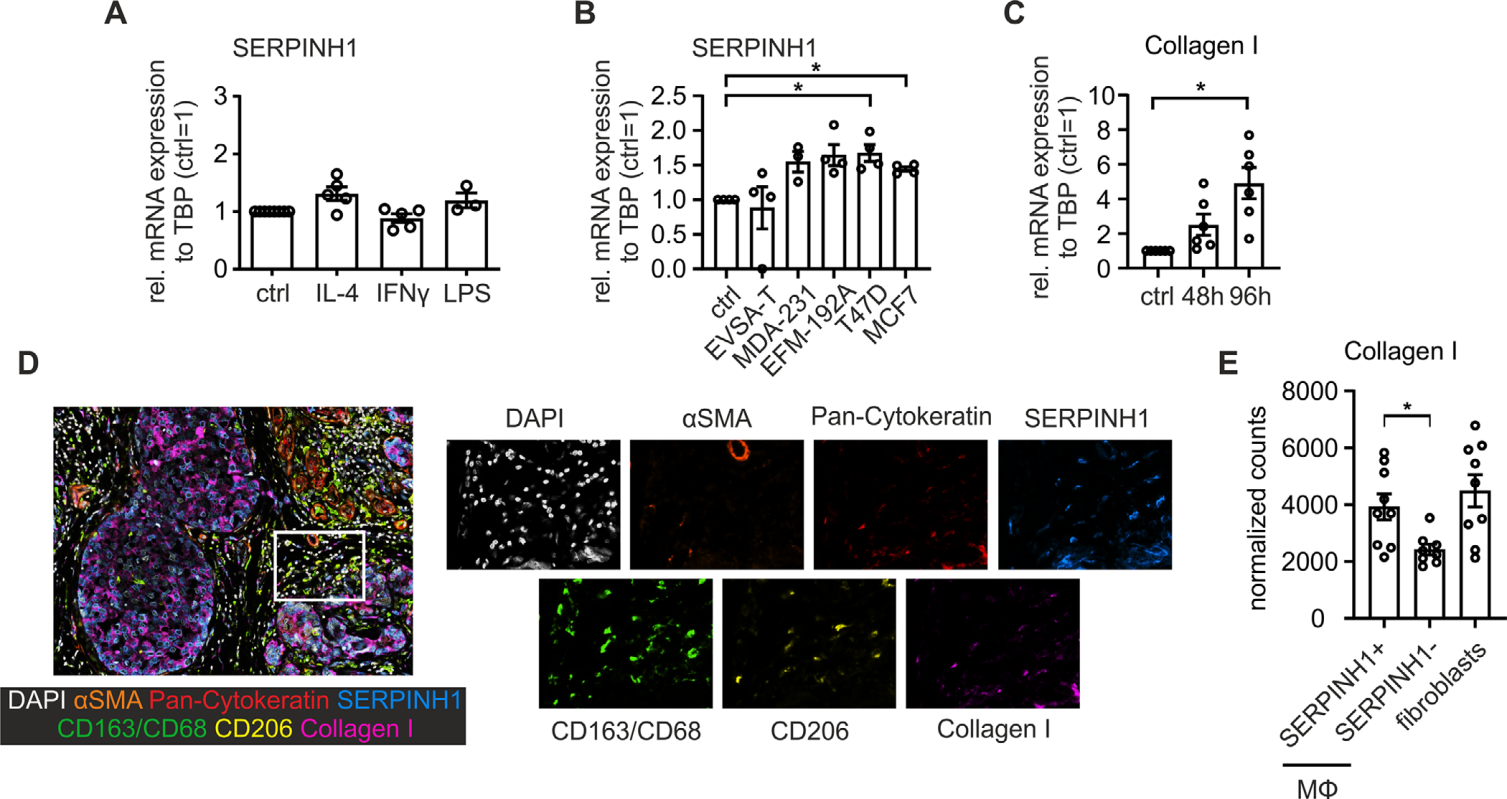
SERPINH1 expression in primary human macrophages and tumor tissue. A, mRNA expression of SERPINH1 in untreated MΦ (ctrl), MΦ treated with IL‐4, IFNγ or LPS (from three to five independent donors). B, mRNA expression of SERPINH1 in untreated MΦ (ctrl) and MΦ after co‐culture with breast cancer cell lines for 96 hours (from four independent donors). C, mRNA expression of cumulative collagen I A1 and A2 expression in untreated MΦ (ctrl) and MΦ after a co‐culture with MCF‐7 cells for 96 hours (from six independent donors). D, Representative immunofluorescence staining of primary breast cancer tissue. Fibroblasts were stained with αSMA (orange), tumor cells with pan‐cytokeratin (red), MΦ with CD163/CD68 (green) and CD206 (yellow). Collagen I expressing cells are shown in magenta, and SERPINH1 is shown in blue. Nuclei were counterstained with DAPI (white). E, Quantitative analysis of collagen I expression in fibroblasts and macrophages with or without the expression of SERPINH1 (n  =  9). The *P*‐values were calculated using either one‐sample *t*‐test for normalized data or unpaired two‐tailed *t*‐test *P < .05

SERPINH1 is the chaperone for the production of collagen I. To analyze which signals might induce its expression in macrophages, we stimulated primary human macrophages derived from Buffy Coats with classical pro‐ and anti‐inflammatory mediators and analyzed SERPINH1 mRNA expression (Figure 5A). SERPINH1 expression was not induced by IL‐4, IFNγ, or LPS stimulation. To test if SERPINH1 expression is regulated by tumor‐specific factors, we performed co‐culture experiments. Indeed, we detected an induction of SERPINH1 after a 96‐hour co‐culture with a number of breast cancer cell lines, which was statistically significant for MCF‐7 and T47D cells (Figure 5B). This was accompanied by higher expression of collagen I, which was calculated by summing up normalized expression values of the isoforms A1 or A2 (Figure 5C), indicating that SERPINH1 expression may mark collagen‐producing macrophages. For further differentiation, we stained FFPE tissue sections from primary human breast tumors of our patient cohort with pan‐cytokeratin for tumor cells, αSMA to identify fibroblasts, and CD163, CD68, and CD206 to mark macrophages (Figure 5D). We then analyzed cells expressing SERPINH1 and collagen I. We were able to separate SERPINH1^+^ and SERPINH1^−^ macrophages, and observed a significant reduction of collagen I expression in SERPINH1^−^ macrophages. Furthermore, collagen I expression in macrophages expressing SERPINH1 was comparable to collagen I expression in fibroblasts (Figure 5E). These results indicate that SERPINH1 expression is regulated by tumor cell‐derived factors and correlates with strong collagen I expression in macrophages.

### 3.4 MORC4 protects macrophages from cell death

**FIGURE 6:**
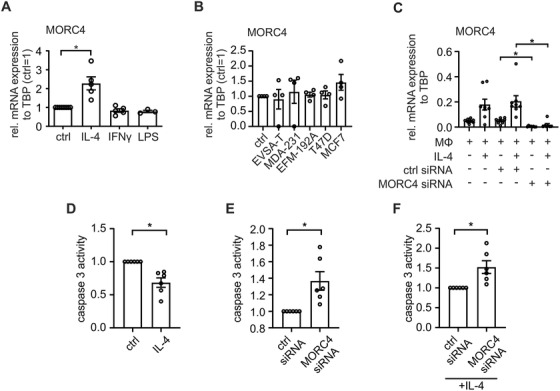
Functional relevance of MORC4 expression in macrophages (A‐C) mRNA expression of MORC4 in MΦ after different stimuli. Data are mean ± SEM. A, MΦ were treated with IL‐4 for 48 hours, LPS or IFNγ for 24 hours (from three to five independent donors). MORC4 mRNA expression was normalized to untreated MΦ (ctrl = 1). B, MΦ were co‐cultured with different breast cancer cell lines for 96 hours (from four independent donors). MORC4 mRNA expression was normalized to untreated MΦ (ctrl = 1). C, MΦ were transfected with ctrl siRNA or MORC4 siRNA for 24 hours followed by IL‐4 stimulation for 48 hours (from eight independent donors). D‐F, Caspase 3 activity assay with MΦ transfected with ctrl siRNA or MORC4 siRNA for 24 hours followed by 48 hours stimulation with IL‐4. To induce apoptosis, cells were incubated with CHX and TNFα for 6 hours. Caspase 3 activity was normalized to either untreated (ctrl) (D) or ctrl siRNA (E and F) transfected MΦ. Time point of analysis was 15 minutes after addition of caspase 3 substrate. Data are mean ± SEM from seven independent donors. *P*‐values were calculated using either one‐sample *t*‐test for normalized data or one‐way ANOVA with Tukey's multiple comparison test *P < .05

MORC4 belongs to a family of proteins associated with DNA damage response, chromatin remodeling, and survival.^34^, ^35^ GSEA indicated altered turn‐over of CD206^+^ and CD206^−^ macrophages in breast tumors. Therefore, we wondered if MORC4 expression affected macrophage proliferation or death. To analyze proliferation of macrophages, we stained primary human breast cancer sections of our cohort using immunofluorescence for co‐localization of Ki67 and MORC4. However, since proliferating macrophages were generally rare, detecting a significant difference in Ki67 expression between MORC4^+^ and MORC4^−^ macrophages was not feasible (data not shown). Next, we explored the involvement of MORC4 in cell survival. Interestingly, MORC4 expression was selectively induced by IL‐4, whereas it was not affected by IFNγ, LPS, or a co‐culture with different breast cancer cell lines (Figure 6A,B). These results are in line with GSEA data from CD206^+^ macrophages, which appeared to share features of macrophages after IL‐4 stimulation (Table S4).

Next, we used siRNA to knockdown MORC4 expression in macrophages after IL‐4 stimulation (Figure 6C). MORC4 expression was significantly reduced both at baseline and after IL‐4 stimulation upon transfection with specific siRNA when compared to control siRNA. Next, we exposed the MORC4 knockdown and control cells to an apoptosis assay with caspase 3 activity serving as the read‐out to assess the role of MORC4 in apoptosis (Figure 6D‐F). Apoptosis was induced by incubating macrophages with CHX and TNFα for 6 hours, followed by the measurement of caspase 3 activity for 1 hour. Caspase 3 activity in IL‐4 treated macrophages was significantly reduced when compared to untreated macrophages, indicating a protective effect of IL‐4 (Figure 6D). Furthermore, there was significantly more apoptosis in MORC4 knockdown macrophages compared to control cells, which was still apparent after the addition of IL‐4 (Figure 6E,F). These results indicate a protective role of MORC4 in macrophages, which can be even further enhanced by IL‐4. Taken together, MORC4 appeared to affect the survival of macrophages and may further affect their proliferation.

## 4 DISCUSSION

Composition of the tumor‐associated immune system is of predictive value, and reactivation of immunity has become a promising tool for treating certain types of cancer. So far a major focus has been on the antitumor activity of lymphocytes, but also innate immune cells determine therapy success.^30^ In this study we identified novel markers for macrophage subtypes, which correlate with survival of breast cancer patients.

In mice, breast tumors are infiltrated with two major macrophage subsets, which, among other markers, can be distinguished by CD206 expression. Our data suggest that human tumors also contain CD206^−^ and CD206^+^ macrophages with distinct properties. Again, as in mice,^36^, ^37^, ^38^ CD206^+^ macrophages are present in the untransformed mammary gland, while an increased CD206^−^ macrophage infiltrate is observed during tumor development, with highest abundance of these cells in invasive tumors. In mouse tumors both macrophage subsets are at least partially derived from blood monocytes,^23^ while CD206^+^ macrophages appear to stem from embryonic sources in the untransformed mammary gland.^36^ Tissue resident CD206+ macrophages in the mouse are principally able to self‐renew by in‐situ proliferation.^36^, ^37^, ^38^ Therefore, the increase in CD206^−^ macrophages may be either related to an increased influx of monocytes, or indicative of altered microenvironmental niches inducing CD206^−^ macrophage differentiation in invasive as compared to noninvasive tumors. CD206^−^ macrophages showed higher levels of both CCR2 and CX3CR1, which are highly expressed by monocytes as compared to mature macrophages.^36^ A third explanation therefore would be that differential CD206 expression levels may reflect distinct differentiation stages, with CD206^−^ having recently differentiated from monocytes and potentially serving as precursors of CD206^+^ macrophages. However, investigations to unravel if human breast CD206^−^ and CD206^+^ macrophages are generated from distinct sources, due to differentiation under different microenvironmental conditions, or if one subset is the precursor of the other, are elusive. In favor of the first explanation, a recent study defined two different monocyte‐derived resident tissue macrophage populations in mouse lungs and other tissues, derived from monocytes and developing independently of each other. Nerve‐associated Lyve‐1^lo^ MHCII^hi^ resident tissue macrophages expressed higher levels of CX3CR1 and were involved in antigen presentation, whereas blood vessel‐associated Lyve‐1^hi^ MHC‐II^lo^ resident tissue macrophages expressed CD206 as well as CD209 and regulated wound healing and tissue repair.^27^ These distinct macrophage populations were also found in a number of other tissues such as heart, adipose tissue, and skin in both, mice and humans.^27^, ^39^, ^40^ Moreover, CD206^+^ macrophages were found in close proximity to the vasculature, particularly lymphatic vessels, in the PyMT mammary carcinoma mouse model.^22^

Our data indicate that both, CD206^−^ and CD206^+^ macrophages subpopulations are also apparent in breast cancer. Chakarov et al discussed the role of Lyve‐1^hi^ MHCII^lo^ CD206^+^ macrophages in protecting tissues from fibrosis.^27^ We found a CD206^+^ macrophage subset expressing SERPINH1 as well as collagen I, suggesting a role in fibrotic processes. While these macrophages shared a niche with fibroblasts, they were associated with positive patient prognosis. It remains to be determined, if collagen‐expressing macrophages directly interact with fibroblasts, which were associated with poor prognosis, thereby suppressing their tumor‐promoting role. A role of activated fibroblasts in cancer progression is now well established. They influence cancer cell metabolism, recruit immune cells and regulate tumor immunity, and modulate the extracellular matrix to limit and/or promote tumor cell invasion.^41^, ^42^ However, the role of collagen‐producing macrophages has not been studied thoroughly yet, although it is known that macrophages can produce a whole repertoire of different collagen species.^43^ SERPINH1, or heat shock protein 47, is required for the correct folding of various types of collagen and has already been connected to cancer progression.^44^, ^45^ One explanation for an opposite effect of SERPINH1 and collagen I production in macrophages and fibroblasts might be that the final assembly of macrophage‐derived collagen is impaired resulting in ER stress, with consequences for macrophage activation.^46^ Interestingly, monocyte‐derived cells with fibrogenic properties, so‐called fibrocytes, have been described before.^47^ While their role in tumor development is unclear, metastatic human breast cancer cells can limit monocyte‐to‐fibrocyte differentiation.^47^ This may suggest a tumor‐suppressive role of fibrocytes in breast cancer as opposed to fibroblasts. Future studies may be warranted to explore the role of collagen‐producing CD206^+^ macrophages, and their relationship to fibrocytes and fibroblasts, in restricting tumor development.

Our findings that CD206^+^ macrophage subsets are associated with good prognosis in breast cancer patients, while MHCII‐expressing CD206^−^ macrophages are associated with a negative prognosis, are controversial based on existing literature for a number of reasons. First, there are multiple studies illustrating the negative influence of CD68^+^ macrophages on overall survival of breast cancer patients.^32^, ^33^, ^48^ In contrast, we did not observe such a correlation in our cohort. Yet, previous studies did not investigate whole tumor sections, but looked at defined areas only. Leek et al used a Chalkley point‐counting method to assess hot spots of high CD68^+^ macrophage infiltration, where patients with tumors showing 12 hot spots or more, had a worse survival probability.^33^ In line with our results, Lee et al aimed at using a similar counting method, but did not observe hot spots. They rather observed a diffuse pattern of CD68^+^ macrophages, which correlated with high tumor grade, tumor necrosis, and large tumor size.^32^ Thus, the method of determining macrophage infiltrates appears to be crucial. We employed a non‐biased approach, which may be more reproducible than defining hot spots. Second, we used a combination of CD68 and CD163 to define macrophages. CD163 is often referred to as a marker of alternatively activated and therefore presumably tumor‐promoting macrophages, which, based on the M1/M2 paradigm, would be expected to also express CD206.^8^, ^10^, ^11^ In pilot experiments, we detected a strong overlap of CD68 and CD163 staining (Figure S9) and did not find a preferential expression of CD206 in either CD68‐ or CD163‐positive macrophages. Indeed, CD163 should be considered a pan‐macrophage marker in humans, which can be induced under M2 conditions, but is still detectable at protein level in other macrophages. This notion has been confirmed by previous studies.^13^, ^49^ Nevertheless, Medrek et al compared the prognostic value of CD68 and CD163 in breast cancer. The authors looked at the localization of the macrophages in the tumor nest compared to stroma, since overall macrophage infiltration was not correlated with patient survival. In their study dense infiltration of CD163^+^ macrophages into the tumor stroma correlated with tumor grade, tumor size, subtypes, and receptor status.^5^ CD68^+^ macrophages were also only present in the tumor stroma and correlated with age of the patient and tumor size.^23^ We did not observe such correlations, which might be due to the fact that we did not separate our specimens in stroma versus tumor and therefore "diluted" the prognostic effect of each macrophage marker. However, since the mentioned studies themselves produced conflicting results, the macrophage content alone may not be a reliable parameter for predicting overall survival in breast cancer. Besides total macrophage infiltration, it has been reported before that anti‐inflammatory (or M2 polarized) macrophages are associated with tumor progression and poor prognosis.^50^, ^51^ Nevertheless, such studies are generally based on single markers defining inflammatory (or M1) versus anti‐inflammatory M2 macrophages, including CD206, which may not be the most valid strategy as suggested by experts in the field.^52^ In our hands, specific macrophage subsets co‐expressing CD206 and SERPINH1 or MORC4 were connected with positive patient prognosis, contradicting the previously mentioned assumption that CD206^+^ M2 macrophages are strictly tumor‐promoting cells. In accordance with our data, Franklin et al reported that in the PyMT mammary tumor model the number of MHCII^hi^ CD11c^hi^ tumor‐associated macrophages increased during the course of tumor progression, while levels of CD11b^hi^ mammary tissue resident‐like macrophages decreased.^23^ CD206 expression was higher in macrophages resembling resident macrophages,^23^ which we also observed in the PyMT model.^21^ Franklin et al also stated that CD206^−^ macrophages promote tumor immune tolerance by modulating the CD8^+^ T cell response.^23^ This observation is also represented in our data. We report a positive correlation of CD206^+^ macrophages, but not of CD206^−^ macrophages, with a number of lymphocyte subsets, including CD8^+^ T cells. Furthermore, CD206^−^ macrophages expressing high levels of MHCII correlated with an unfavorable overall survival prognosis. It is conceivable that macrophages expressing high levels of MHCII interact more frequently with T cells, and therefore may inactivate them, as suggested before in PyMT breast tumors in mice.^24^ MHCII is a well‐ established histological marker. However, a combination of CD206 and a macrophage marker was required to reveal an unexpected negative correlation of MHCII with breast cancer patient survival, since CD206^+^ MHCII^+^ macrophages were not correlated with survival.

Franklin et al also observed a higher turn‐over rate of CD206^−^ macrophages in PyMT tumors,^23^ which does not align with our data in breast cancer patients. GSEA rather indicated a higher proliferation rate and provided first hints for potential protection from cell death in CD206^+^ macrophages (increased expression B‐cell lymphoma 2 [BCL‐2] and induced myeloid leukemia cell differentiation protein [MCL‐1] in the anoikis data set). This might be linked to the expression of MORC4, which was overexpressed in a subset of CD206^+^ macrophages. So far MORC4 expression has been described in breast cancer cells, where it is associated with poor survival. Downregulation of MORC4 suppresses tumor growth by the induction of apoptosis.^35^ The knockdown of MORC4 in human monocyte‐derived macrophages resulted in higher caspase 3 activity, suggesting that MORC4 also regulates the survival of macrophages. Unfortunately, factors such as IL‐4 and CSF1 that induce murine macrophage proliferation did not trigger human monocyte‐derived macrophage proliferation in vitro in our hands.^53^, ^54^ Therefore, we were unable to assess a role for MORC4 in human macrophage proliferation in vitro. An important caveat that may underlie this issue was that we used PBMC‐derived macrophages for our study rather than primary tumor‐associated macrophages (TAMs). The underlying reason was limited availability of material to generate sufficient number of primary TAMs for these studies.

The functional roles of SERPINH1 and especially MORC4 in macrophages require further study, particularly since literature on the function of those proteins in macrophages is not available. Besides limited data on the role of our identified targets in tumor‐associated macrophage biology, our study has other important limitations. First, pre‐selecting CD206^+^ and CD206^−^ macrophages for comparison may have limited the discovery of other prognostic macrophage subsets as compared to other approaches such as single cell RNA‐sequencing. Moreover, only ∼ 50% of our sequenced CD206^+^ and CD206^−^ macrophages were patient‐matched, which probably increased variance in the data set and may have limited the number of identified DEGs. Furthermore, longitudinal studies would have been of interest to analyze to transition in macrophage phenotypes over time. Furthermore, normal tissue used for our analyses was taken from cancer patients. We are aware, that this may have influenced macrophage CD206 expression compared to tissue from healthy individuals. Finally, the breast cancer TMAs we used for validation of markers identified by RNA‐Seq had also considerable limitations. While the datasets included varying degrees of detail concerning a number of clinical parameters, they do not yield longitudinal information concerning differences in macrophage infiltrates over time.

In summary, subpopulations of macrophages that correlate with patient survival reside in breast tumors. To identify these macrophage populations, a frequently used marker, CD206, was in itself not sufficient. In depth analyses of the functional role of the identified macrophage populations and an understanding of how they are generated may improve the development of new therapeutic approaches in breast cancer.

## CONFLICT OF INTEREST

The authors declare that there is no conflict of interest that could be perceived as prejudicing the impartiality of the research reported.

## AUTHOR CONTRIBUTIONS

Elisabeth Strack, Weixiao Sha, Leon Pradel, Bernhard Brüne, and Andreas Weigert conceptualized and designed research; Elisabeth Strack, P. Alexander Rolfe, Tobias Schmid, and Andreas Weigert developed methodology. Elisabeth Strack, Annika F. Fink, and Andreas Weigert performed experiments and acquired data; Elisabeth Strack, P. Alexander Rolfe, Sylvia Hartmann, and Andreas Weigert analyzed and interpreted results; Katrin Bankov, Christine Solbach, and Rajkumar Savai provided technical and material support; Andreas Weigert and Bernhard Brüne supervised research, and all authors participated in writing the manuscript.

## Supporting information

Supporting Information

## Data Availability

The RNA‐Seq data set has been submitted to the NCBI Sequence Read Archive (PRJNA668813). Other data that support the findings of this study are available from the corresponding author upon reasonable request.
