# Supplementary material for: Identification of tumor‐associated macrophage subsets that are associated with breast cancer prognosis
Source: Clin Transl Med. 2020 Dec 6;10(8):e239. doi: 10.1002/ctm2.239 (PMC7719284; doi:10.1002/ctm2.239)
Supplement: Supplementary file 1 — Supporting Information [file CTM2-10-e239-s001.pdf]

Supplemental Data

***Table S1**: Summary of patient cohort*

	**Patients n=41**
Female	
Mean age (range years)	67 (38-84)
**Diagnosis**	
TNBC	6
luminal B	26
luminal A	6
HER2 overexpression	3
**T status**	
T1	9
T2	28
T3	3
T4	1
**N status**	
N0	21
N1	18
Nx	2
**M status**	
M0	40
M1	1
**Therapy**	
Radio	20
Endo	35
Chemo	8
Antibody	1
not indicated	4

Table S2: *FACS antibodies used for sorting and analysis*

antibody	fluorochrome	panel	catalog no	company	RRID
CD1c	PE/Dazzle 594	myeloid	331531	BioLegend	AB_2565292
CD4	PE-CF594	lymph	562316	BD	AB_11154394
CD8	BV786	lymph	563823	BD	AB_2687487
CD14	PerCP-Cy5.5	myeloid	561116	BD	AB_2033939
CD14	APC-H7	lymph	560180	BD	RRID:AB_1645464
CD15	FITC	myeloid/ lymph	560997	BD	AB_10563206
CD16	BV650	myeloid/ lymph	563692	BD	AB_2744298
CD19	APC-H7	myeloid/ lymph	560252	BD	AB_1645468
CD25	BV421	lymph	562442	BD	AB_11154578
CD33	BV510	myeloid/ lymph	563257	BD	RRID:AB_2738102
CD45	AF700	myeloid/ lymph	368514	BioLegend	AB_2566374
CD45	AF700	myeloid/ lymph	560566	BD	AB_1645452
CD45RA	BV605	lymph	562886	BD	AB_2737865
CD45RO	PE	lymph	304205	BioLegend	AB_314421
CD56	PerCP-Cy5.5	lymph	362505	BioLegend	AB_2563914
CD64	BV605	myeloid	740406	BD	AB_2740136
CD80	BV711	myeloid	740801	BD	AB_2740464
CD86	BV711	myeloid	563158	BD	AB_2738036
CD107a	BV421	lymph	562623	BD	AB_2737685
CD124	PE	myeloid	355004	BioLegend	AB_11219385
CD127	BV711	lymph	563165	BD	AB_2738041
CD127	BV711	lymph	351327	BioLegend	AB_11219191
CD141	BV785	myeloid	344115	BioLegend	AB_2572194
CD154	BV421	lymph	310823	BioLegend	AB_10933251
CD163	APC	myeloid	333610	BioLegend	AB_2074533

CD206	PE/Cy7	myeloid	321124	BioLegend	AB_10933248
CD278	PE/Cy7	lymph	313519	BioLegend	AB_10641839
CD279	APC	lymph	329907	BioLegend	AB_940473
CD326	FITC	myeloid/ lymph	324203	BioLegend	AB_756077
HLA-DR	APC/Fire 750	myeloid/ lymph	307658	BioLegend	AB_2572101
LAG3	PerCP-eFuor710	lymph	46-2239-41	eBioscience	AB_2573731
LOX-1	PE	myeloid	358603	BioLegend	AB_2562180
MerTK	BV421	myeloid	367603	BioLegend	AB_2566396
TCRαβ	FITC	myeloid/ lymph	561673	BD	AB_10892811
TCRγδ	APC	lymph	561049	BD	AB_10564072

Table S3: *GSEA report for CD206⁺ macrophages – GO terms*

NAME	ES	NES	NOM p-val	FDR q-val	FWER p-val
GO_BODY_MORPHOGENESIS	-0,53	-2,24	0,00	0,02	0,03
GO_FACE_MORPHOGENESIS	-0,61	-2,23	0,00	0,01	0,03
GO_HEAD_MORPHOGENESIS	-0,55	-2,19	0,00	0,02	0,05
GO_KINESIN_COMPLEX	-0,51	-2,17	0,00	0,01	0,05
GO_REGULATION_OF_POSTSYNAPTIC_MEMBRANE_POTENTIAL	-0,52	-2,05	0,00	0,06	0,18
GO_CARGO_RECEPTOR_ACTIVITY	-0,45	-2,01	0,00	0,08	0,25
GO_CHEMICAL_SYNAPTIC_TRANSMISSION_POSTSYNAPTIC	-0,52	-1,99	0,00	0,08	0,27
GO_ANOIKIS	-0,48	-1,99	0,00	0,07	0,27
GO_REGULATION_OF_NEUROLOGICAL_SYSTEM_PROCESS	-0,45	-1,94	0,00	0,11	0,37
GO_ACID_THIOL_LIGASE_ACTIVITY	-0,51	-1,92	0,00	0,13	0,42
GO_ACETYLGLUCOSAMINYLTRANSFERASE_ACTIVITY	-0,44	-1,91	0,00	0,13	0,44
GO_CILIARY_PLASM	-0,42	-1,85	0,01	0,22	0,56
GO_SCAVENGER_RECEPTOR_ACTIVITY	-0,47	-1,82	0,01	0,25	0,60

Table S4: *GSEA report for CD206⁺ macrophages – GEO dataset accession*

NAME	ES	NES	NOM p-val	FDR q-val	FWER p-val
GSE2935_UV_INACTIVATED_VS_LIVE_SENDAI_VIRUS_INF_MACROPHAGE_UP	-0,29	-1,53	0,01	0,69	0,69
GSE5589_IL6_KO_VS_IL10_KO_LPS_AND_IL10_STIM_MACROPHAGE_45MIN_UP	-0,27	-1,51	0,02	0,73	0,71
GSE25088_CTRL_VS_IL4_STIM_MACROPHAGE_UP	-0,27	-1,49	0,03	0,73	0,73
GSE25123_WT_VS_PPARG_KO_MACROPHAGE_ROSIGLITAZONE_STIM_UP	-0,27	-1,48	0,03	0,76	0,74
GSE8515_CTRL_VS_IL1_4H_STIM_MAC_UP	-0,24	-1,36	0,03	1,00	0,84
GSE25123_CTRL_VS_IL4_STIM_PPARG_KO_MACROPHAGE_DN	-0,27	-1,47	0,04	0,77	0,75
GSE19941_UNSTIM_VS_LPS_STIM_IL10_KO_NFKBP50_KO_MACROPHAGE_UP	-0,29	-1,57	0,04	0,65	0,65
GSE24492_LYVE_NEG_VS_POS_MACROPHAGE_UP	-0,28	-1,47	0,04	0,75	0,75
GSE25088_WT_VS_STAT6_KO_MACROPHAGE_IL4_STIM_DN	-0,34	-1,56	0,06	0,62	0,65
GSE5099_MONOCYTE_VS_ALTERNATIVE_M2_MACROPHAGE_UP	-0,25	-1,32	0,06	1,00	0,86
GSE28783_ANTI_MIR33_VS_CTRL_ATHEROSCLEROSIS_MACROPHAGE_DN	-0,26	-1,36	0,06	1,00	0,84
GSE5589_LPS_VS_LPS_AND_IL6_STIM_IL6_KO_MACROPHAGE_45MIN_UP	-0,24	-1,34	0,07	1,00	0,85
GSE360_T_GONDII_VS_M_TUBERCULOSIS_MAC_UP	-0,26	-1,33	0,07	1,00	0,85
GSE26343_WT_VS_NFAT5_KO_MACROPHAGE_UP	-0,24	-1,30	0,08	0,99	0,87
GSE26343_WT_VS_NFAT5_KO_MACROPHAGE_LPS_STIM_UP	-0,26	-1,35	0,09	1,00	0,84

***Table S5**: Summary of TMA patient cohorts*

	**Relapsed ER+ breast cancer (patients n=54)**	**HER2+ breast cancer (patients n=38)**	**TNBC (patients n=26)**
Female			
Mean age (range years) *	62 (32-82)	58 (26-82)	50 (29-86)
**Diagnosis ***	ER+	HER2+	TNBC
**T status ***			
T1	10	18	4
T2	30	15	22
T3	7	3	-
T4	7	2	-
**N status ***			
N0	5	0	18
N1	31	10	5
N2	16	26	2
N3	2	2	1
**M status ***			
M0	54	38	26
M1	-	-	-
**Stage ***			
1	0	0	18
2	29	9	5
3	25	29	3
**Grade ***			
1	0	0	0
2	12	5	0
3	42	33	26

* All variables determined at diagnosis

Figure S1

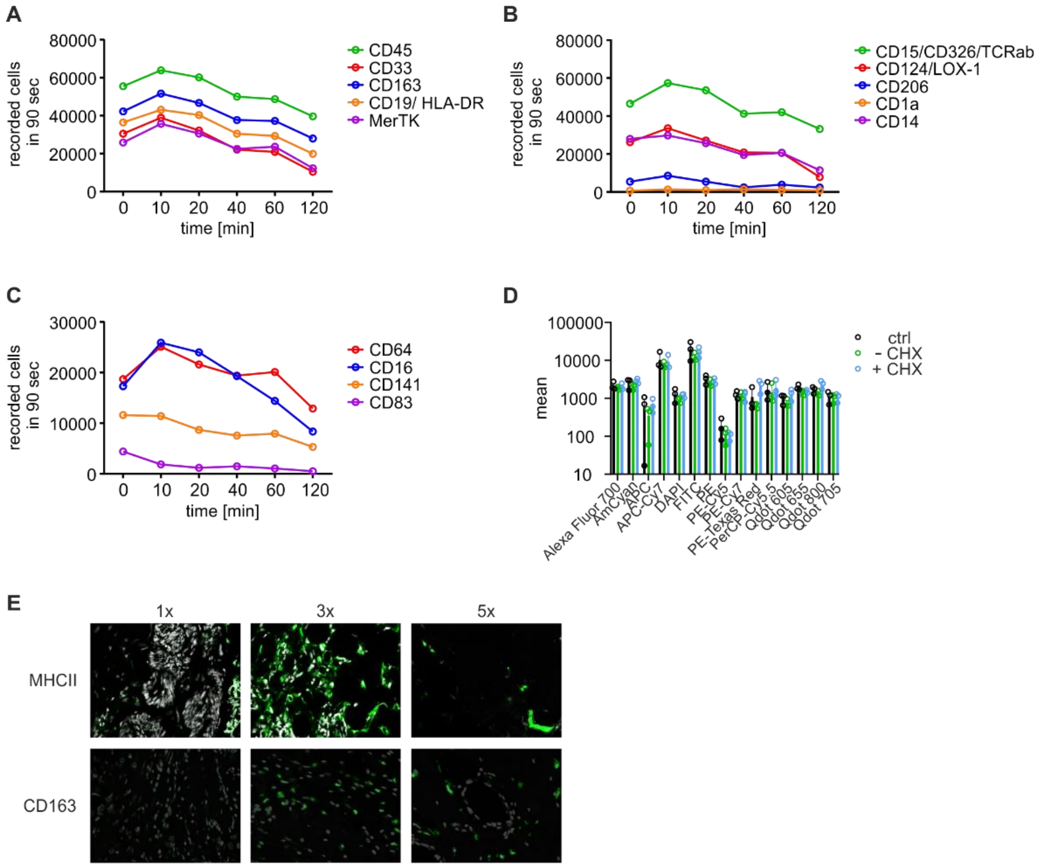

Figure S 1: *Validation of epitope stability for FACS and immunofluorescence . (**A-C)** To study the stability of indicated markers a mixture of PBMCs, macrophages and MCF-7 cells were subjected to the tissue dissociation protocol indicated under Methods for up to 120 min at 37°C. Afterwards samples (n=3) were FACS analyzed. **(D)** Supplement of CHX into storage medium was tested for added epitope stability. Samples were either FACS analyzed directly (ctrl, black, n=3) or incubated for 4 h at 37°C with (+, blue, n=3) or without (-, green, n=3) CHX addition and then FACS analyzed. **(E)** Representative immunofluorescence staining of breast cancer tissue with MHCII or CD163. For the validation of epitope stability and sufficient antigen retrieval in immunofluorescence stainings, FFPE samples were subjected once, three times or five times to heat-induced epitope retrieval and afterwards stained with the*

indicated markers MHCII or CH163 (both green). Nuclei were counterstained with DAPI (white).

Figure S2

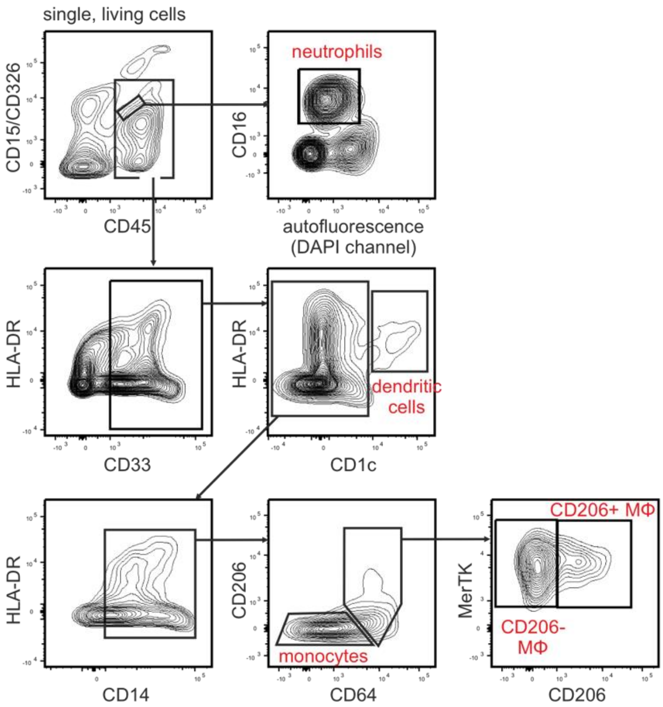

***Figure S2**: Gating strategy of myeloid cells in primary human breast cancer. After identification of single, living cells, immune cells were gated as CD45^{+} cells. CD16 was used to identify neutrophils within CD15^{hi} cells. CD45^{+} cells were further gated for myeloid CD33^{+} cells. These cells were used to identify dendritic cells (HLA-DR^{+}, CD1c^{+}, MerTK^{-}), monocytes (HLA-DR$^{+/-}$, CD14^{+}, CD64^{lo}, MerTK^{lo}), and macrophages (MΦ; MHCII^{hi}, MerTK^{+}), which were further separated by CD206 expression.*

Figure S3

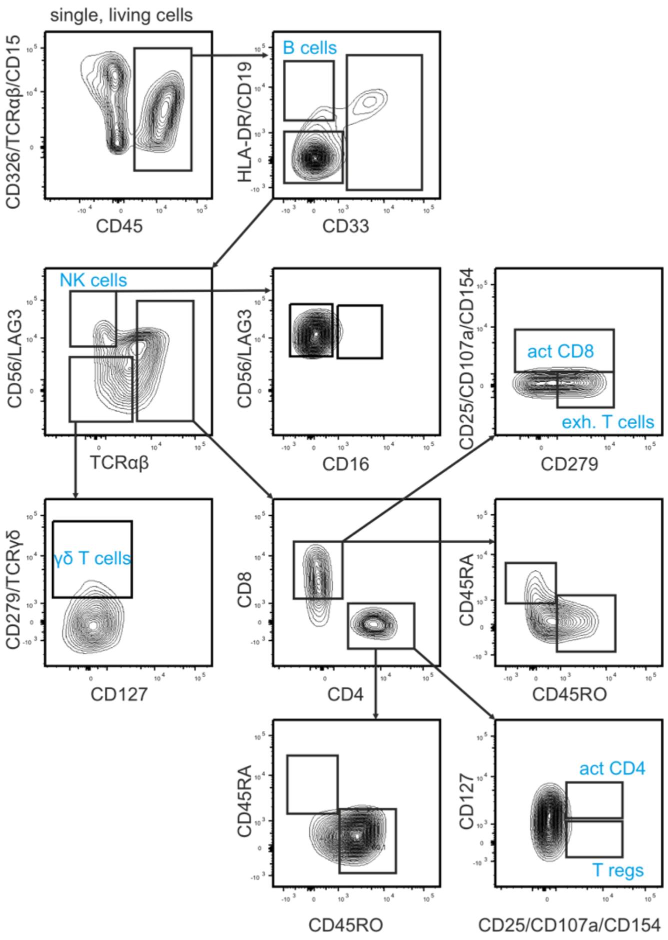

***Figure S3**: Gating strategy of lymphoid cells in primary human breast cancer. After identification of single, living cells, immune cells were gated as $CD45^{+}$ cells. $CD33^{-}$ lymphocytes were separated into B cells ($CD33^{-}$, $CD19^{+}$). Remaining $CD33^{-}$ cells were separated into $CD56^{+}$ cells (NK cells) and $TCR\alpha\beta^{+}$ cells. The $TCR\alpha\beta^{-}$ population was gated for TCRγδ to find γδ T cells. The $TCR\alpha\beta^{+}$ population was further separated into $CD8^{+}$ and $CD4^{+}$ T cells, which were both analyzed for memory (CD45RO) and naïve (CD45RA) populations. Activated $CD8^{+}$ T cells were $CD107a^{+}$ and exhausted (exh.) T cells were $CD279^{+}$. Activated $CD4^{+}$ T cells were $CD127^{+}$ and regulatory T cells (T regs) were $CD127^{low}$ and $CD25^{+}$.*

Figure S4

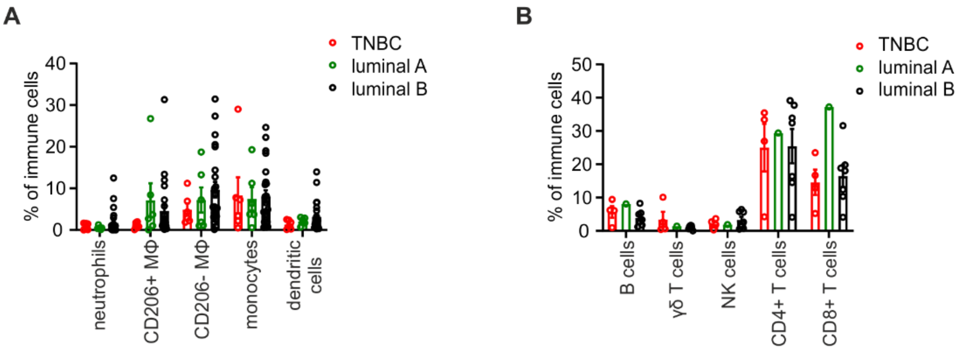

Figure S 4: *FACS based analysis of myeloid (**A**) and lymphoid (**B**) immune cell composition clustered by molecular breast cancer subtype (luminal A (n=6), luminal B (n=26) and TNBC (n=6))*

Figure S5

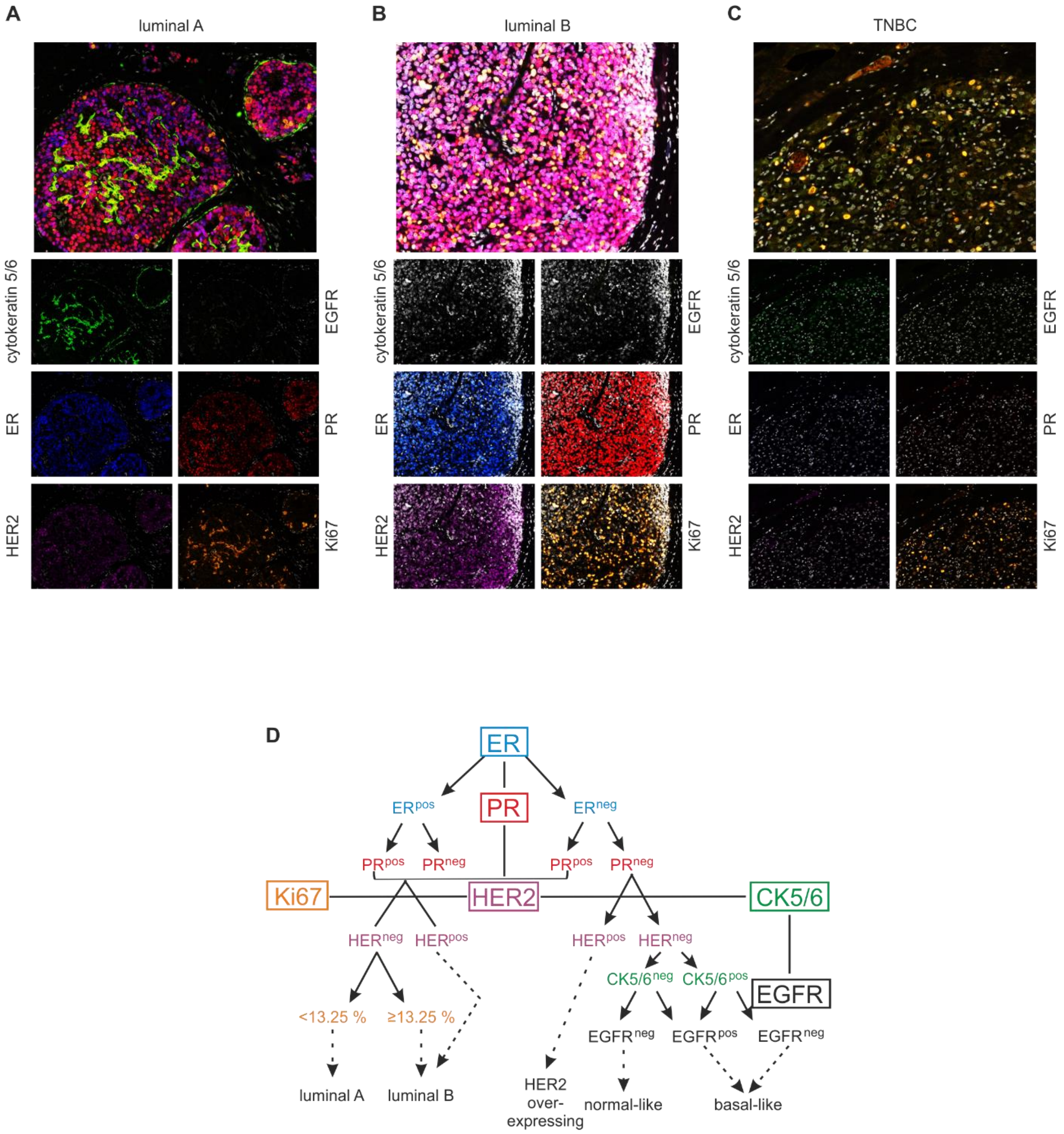

Figure S5: *Molecular profiling of analyzed breast tumors. (**A-C**) Representative immunofluorescence staining of breast cancer subtype luminal A (**A**), luminal B (**B**) and TNBC (**C**). Tumor sections were stained with cytokeratin 5/6 (green), EGFR (yellow), the hormone receptors ER (blue), PR (red), and HER2 (magenta). The proliferation marker Ki67 is depicted in orange and nuclei were counterstained with*

*DAPI (white). (**D**) Overview of molecular profiling scheme connecting all stained markers in A-C.*

Figure S6

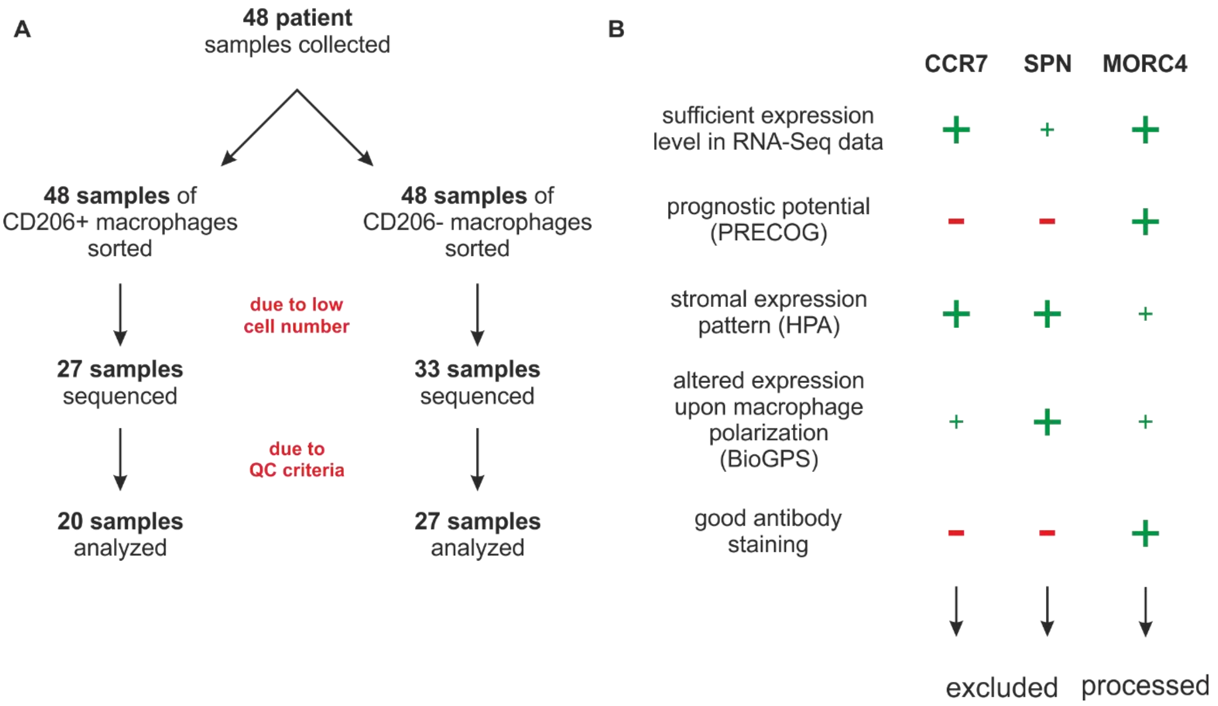

*Figure S6: (**A**) Overview of sample selection and validation. CD206⁺ and CD206⁻ macrophages were simultaneously sorted from 48 patient samples. 27 CD206⁺ macrophage samples and 33 CD206⁻ macrophage samples were sufficient for library preparation. After sequencing 7 CD206⁺ and 6 CD206⁺ macrophage samples were excluded due to low sample quality. (**B**) Example of target selection. DEGs between CD206⁺ and CD206⁻ macrophages were checked for sufficient expression, prognostic potential, stromal expression, altered expression after polarization and availability of antibody. Green plus (+) indicates fulfilled criteria, red minus (–) shows criteria, which are not met.*

Figure S7

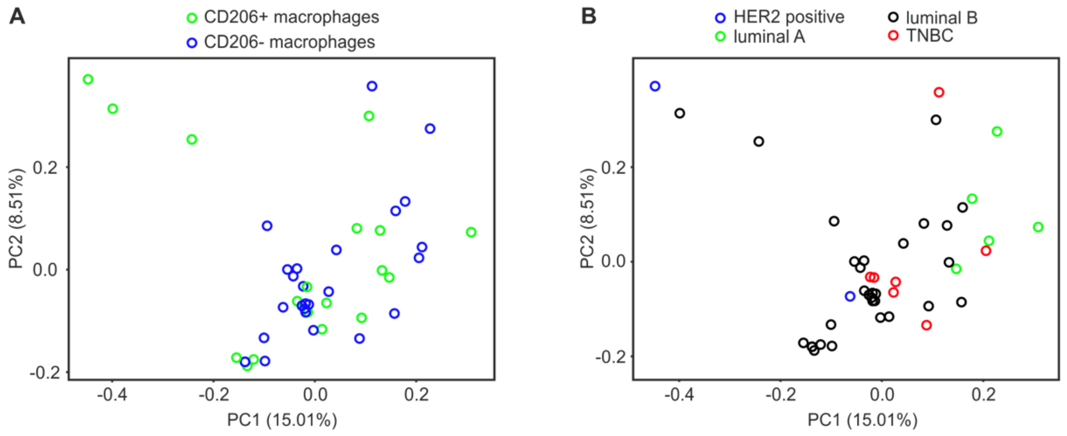

Figure S7: *PCA analysis of macrophages clustered by subpopulation and cancer subtype. (**A, B**) PCA analysis of macrophages sorted from primary breast cancer after quality control. (**A**) Macrophage samples were clustered by CD206 expression. CD206⁺ macrophages are shown in green and CD206⁻ macrophages are depicted in blue. (**B**) Macrophage samples were clustered by the hormone receptor status of tumor sample. Macrophages were sorted from HER2 positive breast tumors (blue), the subtypes luminal A (green), luminal B (black), and TNBC (red). Only samples with an available subtype were included in these plots.*

Figure S8

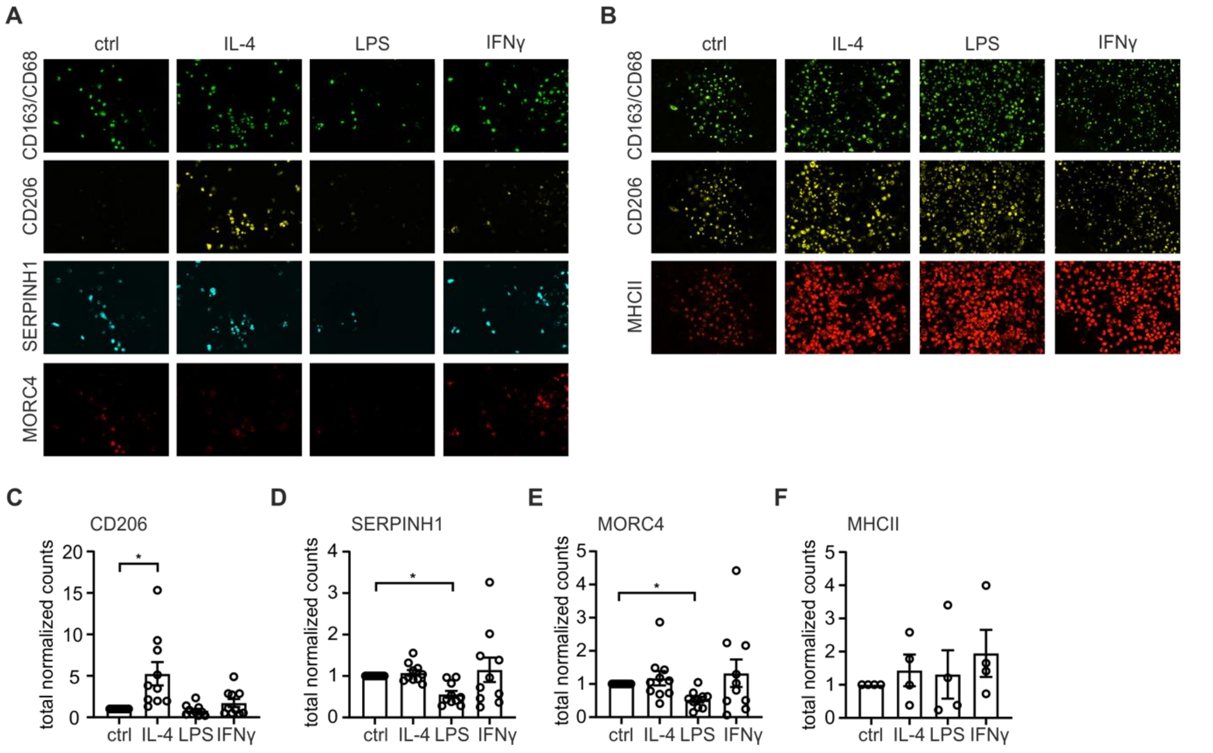

***Figure S8**: (**A-B**) Immunofluorescence staining of in-vitro stimulated MΦ. Untreated MΦ (ctrl) as well as IL-4, LPS and IFNγ stimulated MΦ were stained for CD163/68 (green), CD206 (yellow), SERPINH1 (cyan), and (**A**) MORC4 (red) or (**B**) MHCII (red). (**C-F**) Quantitative analysis of in-vitro stimulated MΦ shown in A and B for the expression of CD206 (n=10) (**C**), SERPINH1 (n=10) (**D**), MORC4 (n=10) (**E**) and MHCII (n=4) (**F**) after IL-4, LPS or IFNγ stimulation compared to untreated MΦ (ctrl). Expression data is normalized to untreated MΦ (ctrl=1). Data are ± SEM.*

Figure S9

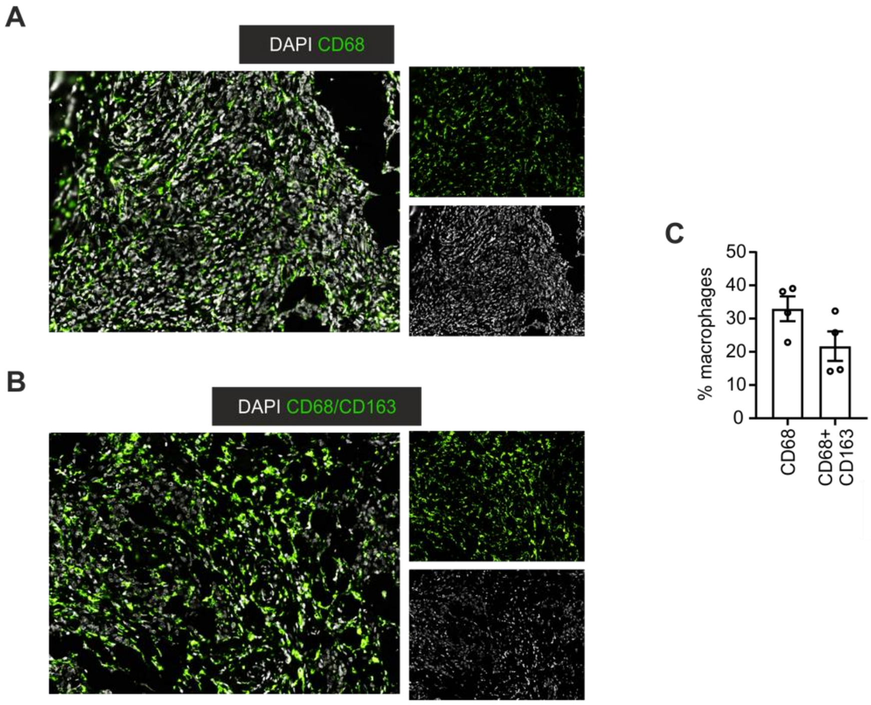

Figure S9*: Histology of macrophage markers. (**A, B**) Representative immunofluorescence staining of one primary breast tumor. Tissue was stained with only CD68 (**A**, green) or with an antibody mix of CD163 and CD68 (**B**, green). (**C**) Quantitative analysis of macrophages stained with either CD68 (n=4) alone or CD68 with CD163 (n=4) in one tumor specimen with 4 independent regions each. Data are ± SEM.*

Figure S10

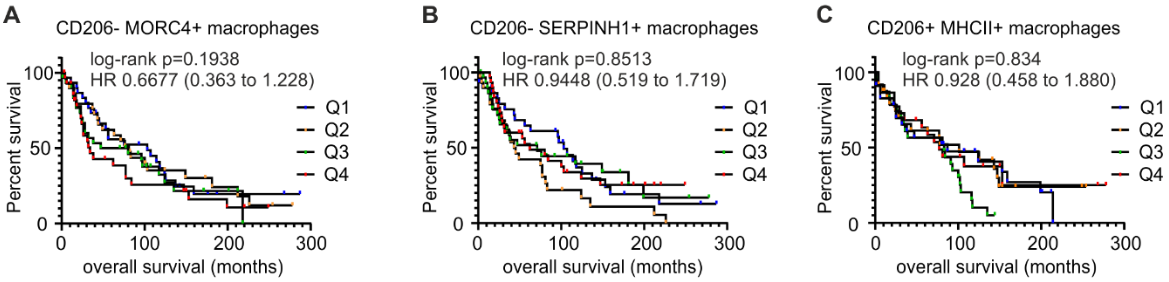

Figure S10**: Macrophage subpopulations and patient survival. Analysis of TMA staining for CD206 in combination with MORC4 (n=154)* ***(A)*, SERPINH1 (n=154)* ***(B)*** *and MHCII (n=94) (C) in invasive breast cancer. Kaplan-Meier estimates analyze the association of indicated cell populations with patient survival. Q1 marks the lowest quartile of indicated cell subtype abundance in the tissues and Q4 the highest quartile (n = 38 in each quartile for D-G, I and K; n = 24 for L). Hazard ratios were calculated between Q1 and Q4..*

Figure S11

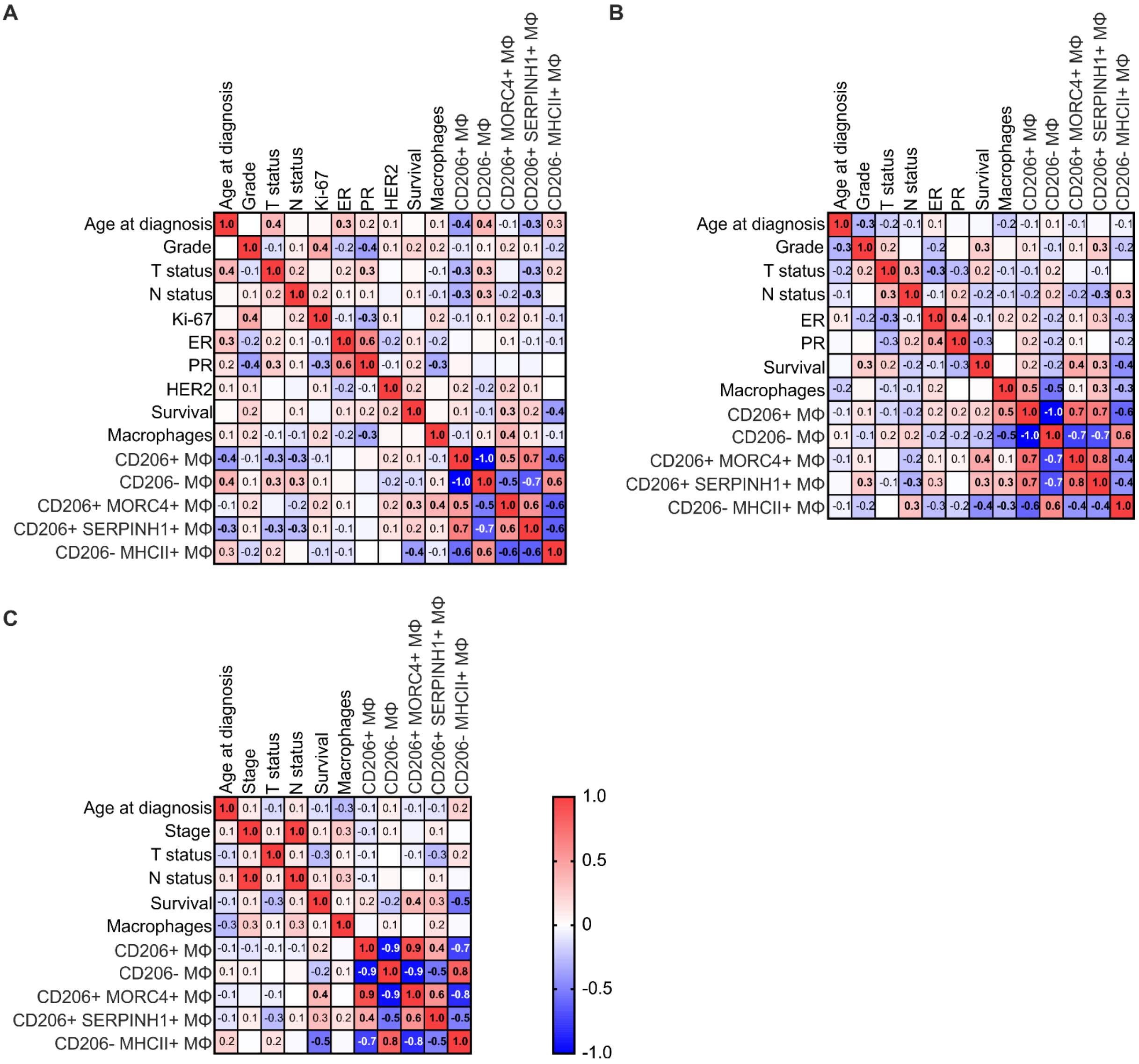

Figure S11: Analysis of TMA staining for CD206 in combination with MORC4, SERPINH1, and MHCII in (A) relapsed ER+ breast cancer (n=54), (B) HER2+ breast cancer (n=38), and (C) TNBC (n=26). Correlation matrices different macrophages populations and clinical parameters available are shown. Positive correlation is indicated in red, negative correlation in blue. Numbers indicate Pearson r-value; bold script indicates statistical significance, p ≤ 0.05.
